# Individual differences in intolerance of uncertainty is primarily linked to the structure of inferior frontal regions

**DOI:** 10.3758/s13415-024-01262-0

**Published:** 2025-01-27

**Authors:** Kenneth W. Carlson, Harry R. Smolker, Louisa L. Smith, Hannah R. Snyder, Benjamin L. Hankin, Marie T. Banich

**Affiliations:** 1 Institute of Cognitive Science, University of Colorado Boulder, Boulder, CO USA; 2 Department of Psychology & Neuroscience, University of Colorado Boulder, D447C Muenzinger Hall, UCB 345, Boulder, CO 80309 USA; 3 Department of Psychology, Brandeis University, Waltham, MA USA; 4 Department of Psychology, University of Illinois at Urbana-Champaign, Champaign, IL USA

**Keywords:** Intolerance of uncertainty, Structural magnetic resonance imaging, Internalizing disorders, Anxious apprehension, Inferior frontal cortex, Orbitofrontal cortex

## Abstract

Increased intolerance of uncertainty (IU), or distress felt when encountering situations with unknown outcomes, occurs transdiagnostically across various forms of psychopathology and is targeted in therapeutic intervention. Increased intolerance of uncertainty shows overlap with symptoms of internalizing disorders, such as depression and anxiety, including negative affect and anxious apprehension (worry). While neuroanatomical correlates of IU have been reported, previous investigations have not disentangled the specific neural substrates of IU above and beyond any overlapping relationships with aspects of internalizing psychopathology. The current study did so in a sample of 42 adults and 79 adolescents, who completed questionnaires assessing IU and internalizing symptoms, and underwent structural MRI. When controlling for internalizing symptoms, across adults and adolescents, specific associations of IU were found with the structure of the inferior frontal cortex and orbitofrontal cortex, regions implicated in cognitive control and emotional valuation/regulation. In addition, in adolescents, associations were observed with rostral middle frontal cortex and portions of the cingulate cortex. No associations were observed with threat-related regions, such as the amygdala. Potential cognitive/emotional mechanisms that might explain the association between individual differences in intolerance of uncertainty and morphology of the inferior frontal cortex are discussed.

**Supplementary information:**

The online version contains supplementary material available at 10.3758/s13415-024-01262-0.

Intolerance of Uncertainty (IU), or distress felt when encountering situations with unknown outcomes, is considered a transdiagnostic feature across internalizing disorders, occurring in generalized anxiety disorder (GAD), major depressive disorder (MDD), and obsessive-compulsive disorder (OCD), amongst others (Gentes & Ruscio, 2011, McEvoy et al., 2019; Dugas et al., 1998). Despite this relationship with multiple forms of psychopathology, the strongest relationships are observed for anxiety disorders (Dugas et al., 2001, 2004), specifically the symptom of worry, a criterial symptom of GAD (Buhr & Dugas, 2006, Osmanağaoğlu et al., 2018). Suggesting an important role for IU in the modulation of internalizing symptoms, reducing IU through targeted interventions focused on reappraisal of emotions associated with uncertainty has shown promising success at reducing symptoms of worry in GAD patients. Moreover, such interventions have been shown to decrease more general symptoms of anxiety and depression, suggesting a multifaceted relationship between IU and symptoms of internalizing disorders that often goes unaccounted for in research on IU (Ladouceur et al., 2000; Boswell et al., 2013). Despite IU’s clinical importance, the neuroanatomical correlates of individual differences in IU after accounting for related yet distinct symptoms of internalizing psychopathology remains unclear. We investigate this issue in the current report in both adult and adolescent samples using a well-validated dimensional model of internalizing symptoms to parse variance that is unique to IU from covariance with other aspects of internalizing psychopathology.

A prominent model of IU and internalizing disorders, known as The Uncertainty and Anticipation Model of Anxiety (UAMA), posits that IU emerges through the interaction of several emotional, cognitive, and neurobiological processes (Grupe & Nitschke, 2013). More specifically, this model posits that IU is supported by 1) increased vigilance and attention towards threat, 2) heightened reactivity to the uncertainty associated with threat, 3) inflated estimates of the probability and cost of threats, 4) deficient learning with regards to what situations and cues are indicative of safety, 5) behavioral and cognitive avoidance of situations associated with threat, and 6) maladaptive control over these processes. This model further links these six processes to alterations within specific neural systems, including brain regions involved in responses to salient and threatening information (amygdala, anterior insula), which are regions regulated by the ventral medial prefrontal cortex (vmPFC), most notably as a result of learning within specific contexts. Also implicated are regions involved in calculating the cost and probability of threat-related outcomes (ventral striatum, orbitofrontal cortex, dorsal medial prefrontal cortex [dmPFC], rostral anterior cingulate cortex [rACC]). Finally, control regions (portions of the frontoparietal network, anterior insula, anterior midcingulate) are involved in regulating such activity and organizing behavior in response to perceived threat (e.g., avoidance of situations perceived as threatening).

Reviews of empirical studies examining the associations of IU with either brain function or structure are in broad agreement with this model (for reviews, see Tanovic et al., 2018; Morriss et al., 2023; and associated special issue). Notably, previous functional imaging studies identify both cortical and subcortical regions associated with intolerance of uncertainty. For example in a meta-analysis of studies examining the processing of uncertainty under different behavioral paradigms, the insula was identified as a common area of activation, whereas a portion of the anterior cingulate and amygdala were identified during paradigms in which individuals need to process uncertainty in the context of associative learning tasks (Morriss et al., 2019). The regions identified in individual studies have ranged across many of the regions discussed in the UAMA model, including dorsolateral and/or medial prefrontal cortex (Schienle et al., 2010; Morriss et al., 2022) to the anterior insula (Gorka et al., 2016) to the amygdala (Morriss et al., 2015).

However, of these studies, relatively few have focused on brain morphology, and few, if any, have examined both adult and adolescent participants as we do in the current study. We have chosen to focus on brain morphology because it has been linked to individual differences in a wide variety of cognitive and emotional characteristics (Kanai & Rees, 2011) and is unlikely to be influenced by an individual’s current state (e.g., attentive, drowsy) or other variables, such as differential strategies, that may impact task-based functional imaging studies. In one previous study of 19 GAD patients and 24 healthy controls, after correcting for multiple comparisons, increased IU was found to be associated with increased volume of the right superior temporal cortex (Hilbert et al., 2015). However, prior to correcting for multiple comparisons, Hilbert & colleagues reported associations of IU with volume of a wide range of regions, including the right striatum, right fusiform cortex, left middle cingulate cortex, and right paracentral lobule, suggesting that associations may be more widespread. In related research in a nonclinical sample of 61 college students, higher levels of IU were associated with increased volume of the striatum, particularly the putamen and to a lesser degree the pallidum (Kim et al., 2017). While the patterns of functional activation are mainly consistent across studies with adult participants compared with adolescent participants, there are hints that IU in adolescents may be differentially associated with ACC activity compared with adults (Krain et al. 2006, 2008). Hence one goal of the current study will be to further examine the morphological characteristics associated with IU in an adult sample while examining how these associations might differ between adults and adolescents.

One common and striking finding across all the prior studies of brain regions associated with IU is that those regions have also been found to be associated with different forms of internalizing psychopathology, including anxiety and depression. Results of a meta-analysis show that individuals with major psychiatric disorders exhibit reduced gray matter volume in the rACC and insula, and those with internalizing disorders specifically exhibit reduced volume of subcortical regions, including the amygdala and hippocampus (Goodkind et al., 2015). Reviews of patterns of functional brain activation in individuals with internalizing disorders yield associations with an overlapping set of regions of the default mode, frontoparietal, limbic, and salience networks (Menon, 2011; Williams, 2017), regions involved in internal thought, emotional processing, and cognitive control respectively. Hence, it is not clear whether relationships previously observed between IU and brain function and structure reflect *specific* associations with IU or whether they alternatively reflect associations driven by the relationship of IU with internalizing psychopathology. Such information could provide a clearer picture about the specific roles of the suite of brain regions frequently implicated across different aspects of internalizing psychopathology. Because the results of previous studies investigating the specific relationship of IU with brain structure are limited and inconsistent, a second focus of the current study is to disentangle IU’s association to brain morphology independent from other related symptoms of internalizing disorders. We address this important issue via the following approach.

In the current study, we assess symptoms of internalizing disorders using a S-1 bifactor modeling (Heinrich et al., 2021) to create latent factor scores to reliably capture common and specific internalizing symptom dimensions (Snyder et al., 2022; explained in more detail in the Supplementary Document S1). The rationale behind this approach is that high scores on commonly used measures of internalizing symptoms, such as derived from the Achenbach internalizing scale (Achenbach et al., 2016), may occur because an individual has many symptoms of anxiety, many symptoms of depression, or some combination of the two. The bifactor approach in contrast, explicitly models the co-occurrence across different types of symptoms into a single common reference factor, while allowing for the quantification of symptom-specific residual factors. While this bifactor model posits four distinct dimensions underlying internalizing symptomology, we specifically focus on two dimensions in the current report: the common internalizing factor and the anxious apprehension-specific factor. Based on previous research and the items that form the reference facet for the common internalizing factor, the common internalizing factor in this S-1 bifactor model likely reflects a general proclivity for psychological distress and motivational anhedonia, whereas the anxious apprehension-specific factor captures the tendency engage in repetitive negative thinking that is often future-focused, about external stressors, and difficult to control (Goring & Papageorgiou, 2008; Watkins et al., 2005). We focus on these factors because previous research suggests that constructs analogous to them (neuroticism for the common internalizing factor and worry for the anxious apprehension-specific factor) are highly associated with IU. Importantly, IU remains relevant to internalizing psychopathology even after accounting for its associations with neuroticism and anxiety (Boelen & Reijntjes, 2009). As such, the psychological processes linking IU to psychopathology appear to be dissociable from co-occurring symptoms of anxiety and depression, highlighting the importance of investigating the neural correlates that are specific to IU over and above these other symptoms.

Several of the six psychological processes outlined by UAMA align with theory regarding the processes underlying common internalizing and anxious apprehension. Specifically, the UAMA processes of increased attention and hypervigilance to threat, increased estimates of threat cost and probability, deficient safety learning, and maladaptive control have all been discussed as being central aspects of either general symptoms of internalizing psychopathology (i.e., common internalizing) or anxious apprehension. As a result, by controlling for common internalizing and anxious apprehension, we are likely removing variance in IU scores that is attributable to the UAMA processes mentioned above. Under this framework, the processes that are then likely unique to IU after accounting for common internalizing and anxious apprehension include a heightened reactivity to threat uncertainty and engaging in behavioral and cognitive avoidance of uncertainty.

To assess IU in the current study, we used the intolerance of uncertainty index (IUI) (Carleton et al 2010), which is divided into two subscales; one focuses on people’s feelings and cognitions about uncertainty (IUI-A), whereas the other examines the degree to which individuals take behavioral measures to reduce uncertainty (IUI-B). Such a division aligns well with the distinctions made by Grupe & Nitschke (2013) between a heightened emotional reactivity to uncertainty and actions taken to avoid situations with high subjective uncertainty.

In addition, we constrain our examination of associations to the specific regions that have been previously implicated as being related to IU or in the processing uncertainty more generally (Wu et al., 2021) and use them as *a priori* regions of interest. These regions include portions of the lateral and medial prefrontal cortex, including rostral and caudal portions of the middle and inferior frontal cortex, both the anterior and rostral cingulate, and the amygdala, insula, and portions of the striatum. A novel aspect of our approach is that we have data across two timepoints, which allows us to create averaged measures to aid in trait-like assessments of both internalizing symptoms and IU that are less likely to be influenced by the particulars of an individual’s state at one particular point in time. Furthermore, our sample has both adults and adolescents, allowing us to examine brain–behavior relationships at two distinct life periods.

Based on the very limited prior literature examining morphological characteristics associated with intolerance of uncertainty, it is difficult to make firm predictions about which regions will be most highly implicated. Nonetheless, we predict, that after accounting for relationships with internalizing psychopathology or worry more generally, the brain regions most likely to show an association with IU are those that Grupe & Nitschke (2013) identified as being involved in evaluative and cognitive aspects of the processing of uncertainty, such as heightened reactivity to uncertainty and avoiding uncertain situations. The regions identified in their model as being associated with these processes include the dlPFC, ACC, OFC, amygdala, insula, and striatum. However, as previous research in an overlapping sample of the adolescents in the current study has found that common internalizing and anxious apprehension-specific symptoms are associated with morphometry of the dlPFC, insula, and striatum (Smolker et al., 2022), it may be that IU in the adolescents will be associated with a more limited set of brain regions, restricted to the ACC, OFC, and amygdala. We also explore whether the different main facets of IU, those related to cognition and feelings compared with those related to behavioral actions, show associations with distinct brain regions. Finally, as some functional studies suggests that the relationship of IU to brain activation in the anterior cingulate may differ between adolescents and adults (Krain et al. 2006, 2008), we anticipate that distinct relationships between IU and morphology of the anterior cingulate between these two age groups may be observed as well.

## Methods

### Participants

Originally 138 adolescents and 79 of their parents were recruited for the Colorado Cognitive Neuroimaging Family Emotion Research (CoNiFER) study designed to examine neural mechanisms underlying cognitive control and emotional processing. Data were collected at two separate timepoints, which occurred approximately 2 years apart. Data collected at each timepoint involved neuroanatomical imaging, multiple functional neuroimaging tasks, cognitive-behavioral tasks, and questionnaires relating to cognition and psychopathology. The current study will focus on structural neuroimaging data and self-report measures related to intolerance of uncertainty and internalizing psychopathology.

Participants were drawn from a community sample originally recruited to participate in two different studies in the GEM (Genes, Environment and Mood) Lab (Benjamin Hankin, P.I.; for details of the two samples and studies, see Hankin et al., 2016; Snyder et al., 2019). These community samples were recruited from the Denver metro area via public schools and using direct mail to target zip codes to maximize demographic and socioeconomic diversity. All participants in the current study were screened to be free of history of neurological insult and magnetic resonance imaging (MRI) contraindicators, but not according to psychopathology status. Only adolescents between the ages of 15 and 24 years were accepted for this study.

Of the 79 adults originally recruited, 58 agreed to return for follow-up testing and imaging. Forty-two of these 58 adults had complete questionnaires and imaging data from both timepoints (which allowed for averaging across timepoints). All 42 adult participants in this final sample are female and serve as the adult sample for the current report. Only five fathers agreed to participate, which did not provide a large enough sample to analyze. Of the 138 adolescents originally recruited, 79 returned for follow-up testing and had complete questionnaire data from both timepoints that allowed for averaging across timepoints and are included in the current report. Demographics of our samples are shown in Table 1.

**Table 1:** Questionnaire scores and demographic information for the adult sample (left) and the adolescent sample (right)

Questionnaire scores	Adults	Adolescents
*Mean* (*SD*)	(N = 42)	(N = 79)
**IUl-A**	27.8 (9.10)	31.0 (11.0)
**IUl-B**	62.4 (20.0)	61.1 (20.6)
**MASQ Anxious Arousal**	19.8 (3.26)	33.5 (6.69)
**MASQ Anhedonia**	51.4 (9.59)	43.5 (7.50)
**MASQ Low Positive Affect**	39.6 (8.24)	27.3 (4.70)
**MASQ Loss of Interest**	11.8 (2.83)	16.2 (3.16)
**Penn State Worry Questionnaire**	39.0 (6.68)	39.2 (7.61)
***Demographics***		
** Avg. age (years)**		
Mean (SD)	49.7 (6.75)	18.2 (1.48)
Median [min, max]	49.4 [36.3, 65.6]	17.9 [15.2, 23.6]
** Race**		
Native American/Alaskan	0 (0%)	3 (3.8%)
Asian	0 (0%)	1 (1.3%)
Native Hawaiian/Pacific Islander	0 (0%)	0 (0%)
Black or African American	2 (4.8%)	6 (7.6%)
White	37 (88.1%)	58 (73.4%)
Other/Not Reported	1 (2.4%)	0 (0%)
Mulitracial	2 (4.8%)	11 (13.9%)
** Ethnicity**		
Hispanic/Latino	3 (7.1%)	14 (17.7%)
Non-Hispanic/Latino	38 (90.5%)	65 (82.3%)
** Gender**		
Male	0 (0%)	39 (49.4%)
Female	42 (100%)	40 (50.6%)

*Note.* Unless otherwise noted, mean values are presented followed by the standard deviation in parentheses. IUI = Intolerance of Uncertainty Index; MASQ = Mood and Anxiety Symptom Questionnaire

With regards to psychotropic medications, they were currently being used by four adults (2: fluoxetine, 2: venlafaxine) and 11 adolescents: (3: fluoxetine, 3: lisdexamfetaimine, 1: escitalopram, 1: sertaline, 2: multiple prescriptions (1: fluoxetine and dextroamphetamine, 1: fluoxetine and amitriptyline). One could not recall.

Informed consent was obtained from all adult participants, and adolescent assent and parental consent were obtained for all participants younger than 18 years. All procedures were approved by the University of Colorado Institutional Review Board Protocol 15–0375.

### Intolerance of uncertainty

Levels of IU were measured with the intolerance of uncertainty index (IUI) developed by Gosselin & colleagues (2008; Carleton et al., 2010). The IUI consists of 45 items, all of which are rated according to a five-point Likert scale (“not at all characteristic of me” to “entirely characteristic of me”). Examples of these questions include “Waiting periods are unbearable for me when I do not know what is going to happen” or “I have difficulty dealing with the possibility that something unexpected may occur.” Items are divided into two subscales. The first, IUI-A, focuses on the degree to which people find uncertainty to be unacceptable and threatening and consists of 15 items. The second subscale, IUI-B, examines the effects of that mindset, such as the degree to which individuals worry, seek reassurance, avoid certain situations, or take behavioral measures to reduce uncertainty and consists of 30 items. This measure has been found to have high reliability (α = .86 to .96) and good test–retest reliability (*r* = .66 to .76) (Gosselin et al., 2008).

### Assessments of internalizing psychopathology

#### Self-report questionnaires

##### Mood and Anxiety Symptoms Questionnaire (MASQ)

The MASQ (Watson et al., 1995) is a 39-item scale evaluating symptoms of anxious arousal (17 items, e.g., “Startled easily”; “Was trembling or shaking”) and two anhedonic depression subscales: low positive affect (14 items, e.g., “Felt like nothing was very enjoyable”) and loss of interest (8 items; e.g., “Felt like there wasn’t anything interesting or fun to do”). Participants are asked to rate each item on how much they have felt or experienced it during the past week, from 1 (“not at all”) to 5 (“extremely”). Higher scores on the MASQ indicate greater levels of symptomology. It has good internal consistency, test–retest reliability, and convergent and discriminant validity in relation to depression and anxiety disorders in relevant samples (Watson et al., 1995)

##### Penn State Worry Questionnaire (PSWQ)

Levels of worry were assessed with the PSWQ (Meyer et al., 1990), a 16-item self-report questionnaire assessing tendency to worry (e.g., “My worries overwhelm me.”). It has been found to have good internal consistency, test–retest reliability, and convergent and discriminant validity in relation to anxiety disorders (Brown et al., 1992; Molina & Borkovec, 1994). Higher scores on the PSWQ indicate higher levels of worry.

#### Latent variable modeling

Research has suggested that aspects of internalizing psychopathology can be characterized by a bifactor model, in which there is a common factor that represents symptoms that occur across different aspects of internalizing disorders (i.e., depression, anxiety), and then more specific symptom factors (e.g., anxious apprehension) (Snyder et al., 2022). Specifically, Snyder & colleagues (2022) demonstrated that items from the MASQ and PSWQ are best characterized by a four-factor S-1 bifactor solution, including a common factor reflecting motivational anhedonia and distress (MASQ loss of interest subscale items), as well as low positive affect (MASQ low positive affect subscale), anxious arousal (MASQ anxious arousal subscale), and anxious apprehension (PSWQ)-specific residual factors, with all factors specified as orthogonal (Chen et al., 2012). Notably, when a symmetrical bifactor model with a MASQ loss of interest subscale (which captures motivational anhedonia and distress) specific factor was initially tested, this specific factor had nonsignificant item loadings and factor variance; these items were completely subsumed by the common factor. Thus, the MASQ loss of interest subscale became the reference facet in the final S-1 bifactor model, such that the common factor is defined by these items and thus captures motivational anhedonia and distress. This factor structure has been replicated across multiple samples, with evidence that each factor has distinct diagnostic and neural correlates (Banich et al., 2020; Smolker et al., 2022, 2023; Snyder et al., 2022).

We applied this S-1 bifactor model discussed in Snyder et al. (2022) to the adolescent and adult samples through confirmatory factor analyses. Factor scores were computed separately for each sample and at each timepoint, resulting in a total of four sets of factor scores. However, because intolerance of uncertainty has been linked mainly to the cognitive aspects of anxiety (Anxious Apprehension), but not the physiological aspects (Anxious Arousal, i.e., panic), nor to Low Positive Affect, only the Common Internalizing factor and the Anxious Apprehension-specific factor scores are used in this report. A more detailed description of this bifactor model can be found in Supplementary Materials (S1), including information on model fit.

#### Structural MRI acquisition

Structural MRI data were acquired at the Intermountain Neuroimaging Consortium located at the University of Colorado Boulder using a Siemens 3-Tesla PRISMA MRI scanner for all but 9 adults and 16 adolescents for whom data were collected on a previous version of the same magnet (TIM TRIO). For all participants, a 32-channel head coil was used to collect a high-resolution T1-weighted Magnetization Prepared Gradient Echo (MPRAGE) sequence with the following parameters: number of slices = 224; repetition time (TR) = 2400 ms; echo time (TE) = 2.07 ms; flip angle = 8°; field of view (FoV) = 256 mm; and a voxel dimension = .8 x .8 x .8 mm.

#### Gray matter morphometry preprocessing

T1-weighted structural images were brain-extracted by using a hybrid watershed/surface deformation procedure (Ségonne et al., 2004), followed by a transformation into Talaiarch space, intensity normalization (Sled et al., 1998), tessellation of the gray/white matter boundary (Fischl et al., 2001), and surface deformation along intensity gradients to optimally differentiate gray matter, white matter, and cerebral spinal fluid (CSF) boundaries (Dale et al., 1999; Fischl & Dale, 2000). The resulting segmented surfaces were registered to a standard spherical inflated brain template (Fischl et al., 1999a, b), parcellated according to gyral and sulcal structure (Desikan et al., 2006; Fischl et al., 2004), and then used to compute a range of surface-based measurements, including cortical volume, surface area, and thickness.

Prior to running surface-based analyses, data quality assurance was checked by using FreeSurfer’s standard quality assurance tools (https://surfer.nmr.mgh.harvard.edu/fswiki/QATools), including checking for volume-based statistical outliers (±3 standard deviations) of subcortical regions of interest (ROI) segmentation data, signal-to-noise ratio, and mean and standard deviation of white matter intensity. Data quality for all 121 participants were deemed acceptable as SNR for the adult sample ranged between 16 and 24 (*M* = 19.8, *SD* = 2.05) for timepoint 1 and timepoint 2 (timepoint 2; *M* = 19.6, *SD* = 1.84); white matter intensity for timepoint 1 ranged between 103 to 107 (*M* = 105, *SD* = 0.754) and 104 to 107 for timepoint 2 (*M* = 105, *SD* = 0.724). In the adolescent sample, SNR ranged from 16 to 25 for timepoint 1 (*M* = 19.4, *SD* = 2.04) and 16 and 24 for timepoint 2 (*M* = 19.4, *SD* = 1.88); white matter intensity for the adolescent sample at timepoint 1 ranged from 105 to 108 (*M* =106, *SD* = 0.718), and for timepoint 2, it ranged from 105 to 108 (*M* = 106, *SD* = 0.669). A co-author of this report (HRS) performed visual inspection of the subcortical segmentation and whole brain cortical surfaces to ensure that the distinct subcortical structures appeared to be properly segmented and that the white and pial surfaces aligned with the distinction between cortical gray and white matter visible when viewing the T1 structural images. Regions of interest analyses were performed in fsaverage7 space (163,842 vertices per hemisphere).

#### Linear regression analysis

Analyses were performed with the ROI that have been shown in previous work to yield associations with IU (see *Introduction*). In our ROI approach, we first extracted total volume, surface area, and thickness from the relevant cortical ROIs as defined by the Desikan-Killany atlas (Desikan et al., 2006) (see Supplementary Fig. S1 and Fig. S2 for ROIs utilized in current study). For the noncortical ROIs, the “Aseg Atlas” was used to define and extract volumes (Fischl et al., 2002). The regions examined fell into 5 cortical regions and 4 subcortical regions. The cortical groups were: inferior frontal gyrus (pars triangularis, pars opercularis, and pars orbitalis); dorsolateral prefrontal cortex (caudal middle frontal, rostral middle frontal); orbitofrontal cortex (medial, lateral); cingulate cortex (rostral anterior, caudal anterior, posterior cingulate, isthmus); and the insula. The subcortical regions were the amygdala, putamen, caudate, and pallidum. Morphological characteristics of these regions were calculated separately for the right and left hemispheres. Our analyses focus on metrics of thickness and surface area for cortical regions, and volume for the subcortical regions. As was the case with all other metrics used in our models, we computed neuroanatomy metrics by averaging ROI values across timepoints 1 and 2.

To test for associations between gray matter morphometry and IUI, multiple regression models were run in Rstudio 2022.02.1+461 “Prairie Trillium” Release (8aaa5d470dd82d615130dbf663ace5c7992d48e3, 2022-03-17) for macOSMozilla/5.0 (Macintosh; Intel Mac OS X 12_6_1) AppleWebKit/537.36 (KHTML, like Gecko) QtWebEngine/5.12.10 Chrome/69.0.3497.128 Safari/537.36 using the ‘lm()’ function in the ‘stats’ package version 4.1.3 (Chambers, 1992; Wilkinson & Rogers, 1973). In all models, morphometric measures of specific ROIs were the dependent variable and were predicted by either IUI-A or IUI-B with the demographic variables of age and gender (for adolescent models only) also included. We ran two sets of regressions. One set was designed to determine how much IUI is associated with morphology above and beyond internalizing psychopathology. In these models, the Common Internalizing factor score as well as Anxious Apprehension-Specific Factor score were included in the model as covariates. While IUI-A and IUI-B are correlated with each of these factor scores (Table 2), they are not highly collinear in the adults (*r* = .29 to .56), although they are more so in the adolescents (*r* = .49 to .69). As such, the residual variance accounted for by an IUI measure in regression models that also contain the Common Internalizing and Anxious Apprehension-Specific factors may be limited in the adolescent sample. The other set of regressions had just one psychologically related predictor in the model—an IUI scale—and was designed to reveal the relationship of IU with brain morphology without consideration of any potential overlap of IU with other aspects of internalizing disorders.

For surface area and thickness models, the mean surface area or mean thickness of the hemisphere in which a morphological feature was located were used as covariates, whereas for volumetric measures, total estimated intracranial volume (EIV) was used as a covariate. When such covariates (e.g., right hemisphere mean thickness) have a significant association in a given model, and there is also a significant association with a specific morphological characteristic (e.g., right anterior cingulate thickness), it suggests that it is the relative size/value of that specific morphological characteristic, not its raw numerical value, that has a predictive association. For example, if in our model greater levels of IUI as well as the overall measure of the relevant morphological characteristic (e.g., mean hemispheric surface area) are significant, it would suggest that individuals with a relatively greater value of that morphological characteristic have higher levels of IUI. Because some of our data were acquired before the upgrade of our scanner, we also included a code for scanner type as a covariate (TRIO = 0, PRISMA = 1).

We ran models for the adolescent sample as a whole with gender as a covariate and interaction term with the anxious apprehension-specific score in our model, gender by Anxious Apprehension, because females had significantly higher Apprehension-Specific Factor scores than males (*t* = 4.805, *df* = 74.765*, p* < .001*)*. Significant gender interactions were observed, but not for the models where IUI was a significant predictor; hence they are not discussed further. Additional exploratory models were run to examine gender-specific associations within each gender and these results are briefly reviewed in this manuscript.

Because using regression is subject to influence from outliers, a Cook’s distance approach was used in all analyses by utilizing the ‘cooks.distance’ function in base r (Robinson, 1984; Fox, 1997; Williams, 1987). All participants whose scores were greater than 4 times the mean of the samples overall Cook’s distance were removed from analyses, and models were rerun. These results are reported below. It is important to highlight that using a Cook’s distance approach will produce analyses with differing sample sizes because not all measures have the same participants identified as outliers. The removed participants range from one to four participants in the adult sample and zero to seven participants for the adolescent sample.

To be conservative in our conclusions within each of the three sets of morphometric analyses (volume, surface area, and thickness), we used the false-discovery rate (FDR) to account for the multiple brain regions investigated. We corrected for the 24 cortical brain regions designated as ROIs and separately for the 8 subcortical regions. These corrections were done using the ‘p.adjust’ function in r (‘stats’ package version 4.1.3; Benjamini & Hochberg, 1995). All results in IUI models that pass FDR corrections and their corrected *p*-values are reported.

It was not possible to precisely calculate the power required for the present study. However, we have good reason to believe that the study is adequately powered. While some researchers argue that sample sizes of approximately 80 or more are required for detecting brain–behavior relationships (Grady et al., 2021), these estimates are derived from examining relationships with measures of functional brain activation. In contrast, metrics of brain morphology on an individual level have been found to be quite robust (Knussmann et al., 2022), and regions that are most variable (e.g., frontal pole, temporal pole) and are not regions that served as ROIs in the present study.

Moreover, as discussed by Baker et al. (2021), it is not only the reliability of the brain data that is important for determining brain–behavior relationships but also the reliability of the behavioral measure. The metric of intolerance of uncertainty that we employed has been shown to have good psychometric properties. Importantly, we collected both brain and behavioral data over two timepoints 2 years apart and averaged across them. As such, the measures we obtained on both brain and behavior are likely to reflect stable individual characteristics that are not specific to the collection timepoint. Moreover, we have found that collection of functional data, which is less stable at the individual level than neuroanatomical data, over two similarly spaced timepoints allows for robust associations of brain–behavior relationships with similar sample sizes (Smith et al., 2023).

## Results

### Correlations

The rationale for averaging across timepoints is that the average score would better reflect individual characteristics of an individual (i.e., trait characteristics) that are independent of the given state or time at which they were acquired. If indeed our measures are picking up on stable individual traits, we would expect that these values should be consistent across timepoints. Moreover, we would expect our predictor variables should show correlations consistent with those reported in the prior literature. As described below, both of these expectations were met.

#### Across Timepoints

To assure that averaging across timepoints to extract more trait-like estimates is reasonable, we ran Pearson correlations for our measures across timepoint 1 and timepoint 2, which as described earlier were collected approximately 2 years apart. As shown in Table S1, all our measures were significantly correlated across timepoints (*p* < .05). For measures related to IU and psychopathology, correlations across the two timepoints ranged from .61 to .84 in the adult sample and from .46 to .59 in the adolescent sample, consistent with our assumptions and expectations. With regards to brain measures (i.e., hemisphere mean surface area, thickness, and EIV) correlations across the two timepoints ranged from .78 to .98 in the adult sample and between .81 to .99 in the adolescents (Table S2).

#### Amongst predictor variables

We explored whether the relationships in our samples between our measures of psychological symptoms (averaged across timepoints) were as expected. Because IU has been found to be highly associated with internalizing disorders and anxiety (Gentes & Ruscio, 2011; McEvoy et al., 2019; Dugas et al., 1998), we tested to see whether such relationships were observed in our sample. As expected, both IUI-A and IUI-B were positively correlated with our measures of internalizing psychopathology (Common Internalizing Factor) as well as our measure of anxiety (Anxious Apprehension-Specific Factor) for both adults and adolescents (Table 2). However, they were not highly co-linear (most correlations having *r* < .8). As is standard for the S-1 bifactor model, the common and specific factors are specified to be orthogonal. However, because extracted factor scores do not perfectly reflect the true latent factors, it is important to verify that the factor scores remain relatively orthogonal. As desired, the Common Internalizing and Anxious Apprehension-Specific Factor were weakly and not significantly correlated. Supplementary Table S3 provides information about these variables and their relationship to whole-brain measures (e.g., cortical thickness).

**Table 2:** Correlations between measures related to intolerance of uncertainty and traits related to psychopathology

Measures				
*Adults*				
	IUI-A	IUI-B	Common internalizing factor	Anxious apprehension specific factor
IUI-A	-			
IUI-B	0.81****	-		
Common Internalizing factor	0.29*	0.40**	-	
Anxious Apprehension Specific factor	0.56***	0.53***	0.18	-
*Adolescents*				
IUI-A	-			
IUI-B	0.88****	-		
Common Internalizing factor	0.61****	0.69****	-	
Anxious Apprehension Specific factor	0.49****	0.55****	0.23*	-

*Note.* IUI-A = Intolerance of Uncertainty Index - subscale A, IUI-B = Intolerance of Uncertainty Index - subscale B *p* < .0001; ****p < .001; ***p < .01; **p < .05; *p < .1

### Linear regressions

**Fig. 1:**
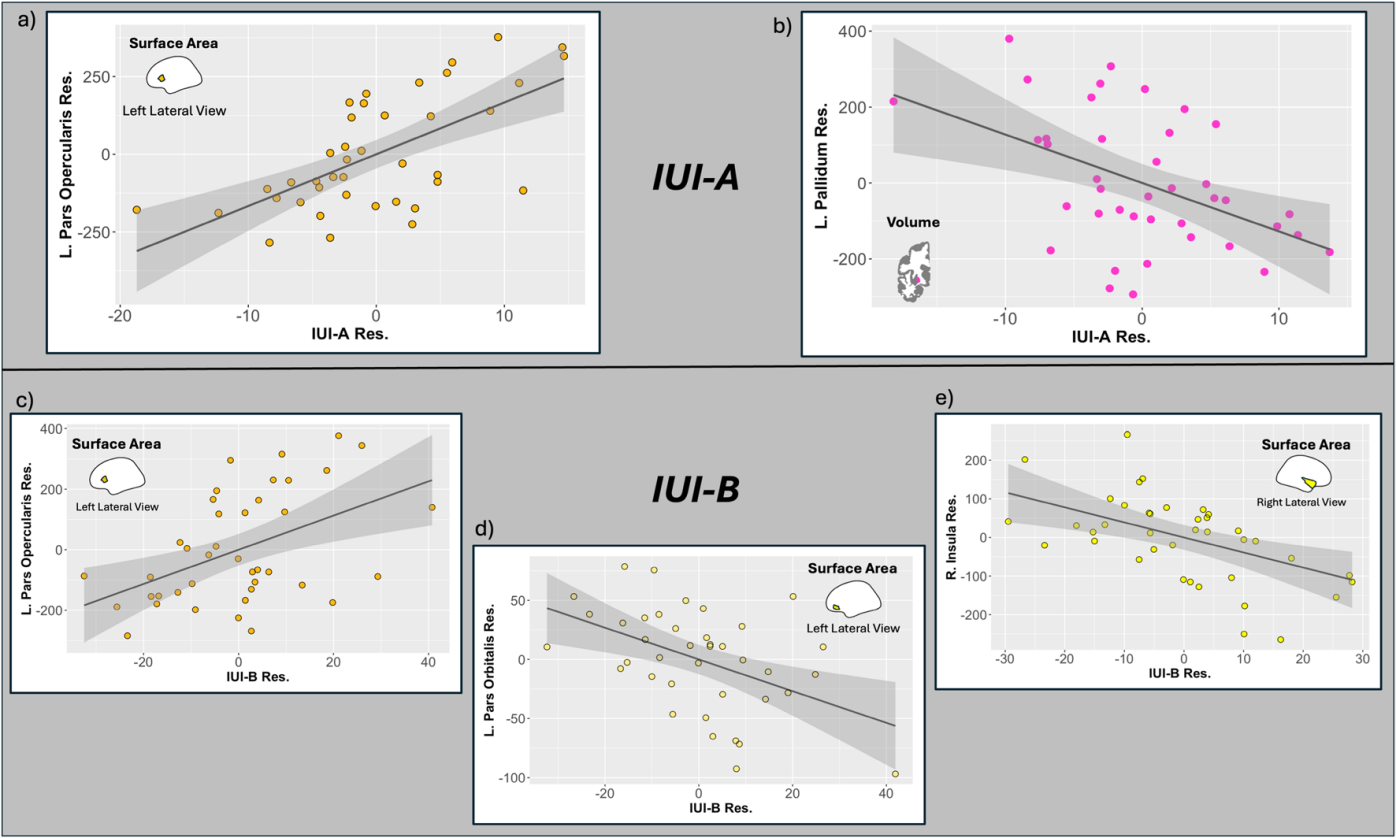
Residual associations in adults of IUI-A and IUI-B with characteristics of brain morphology (Top) The associations between the residuals of IUI-A and the residuals of the (**a**) left pars opercularis surface area and (**b**) left palladium volume. (Bottom) The associations of IUI-B residuals and the residuals of the surface area of both (**c**) left pars opercularis, (**d**) the left pars orbitalis, and (**e**) the right insula. The grey-shaded regions on the graphs are the 95% confidence intervals. Residuals were calculated controlling for Common Internalizing, Anxious Apprehension, Age, Scanner Code, and Overall Brain Characteristic (e.g., Hemispheric Surface Area)

**Fig. 2:**
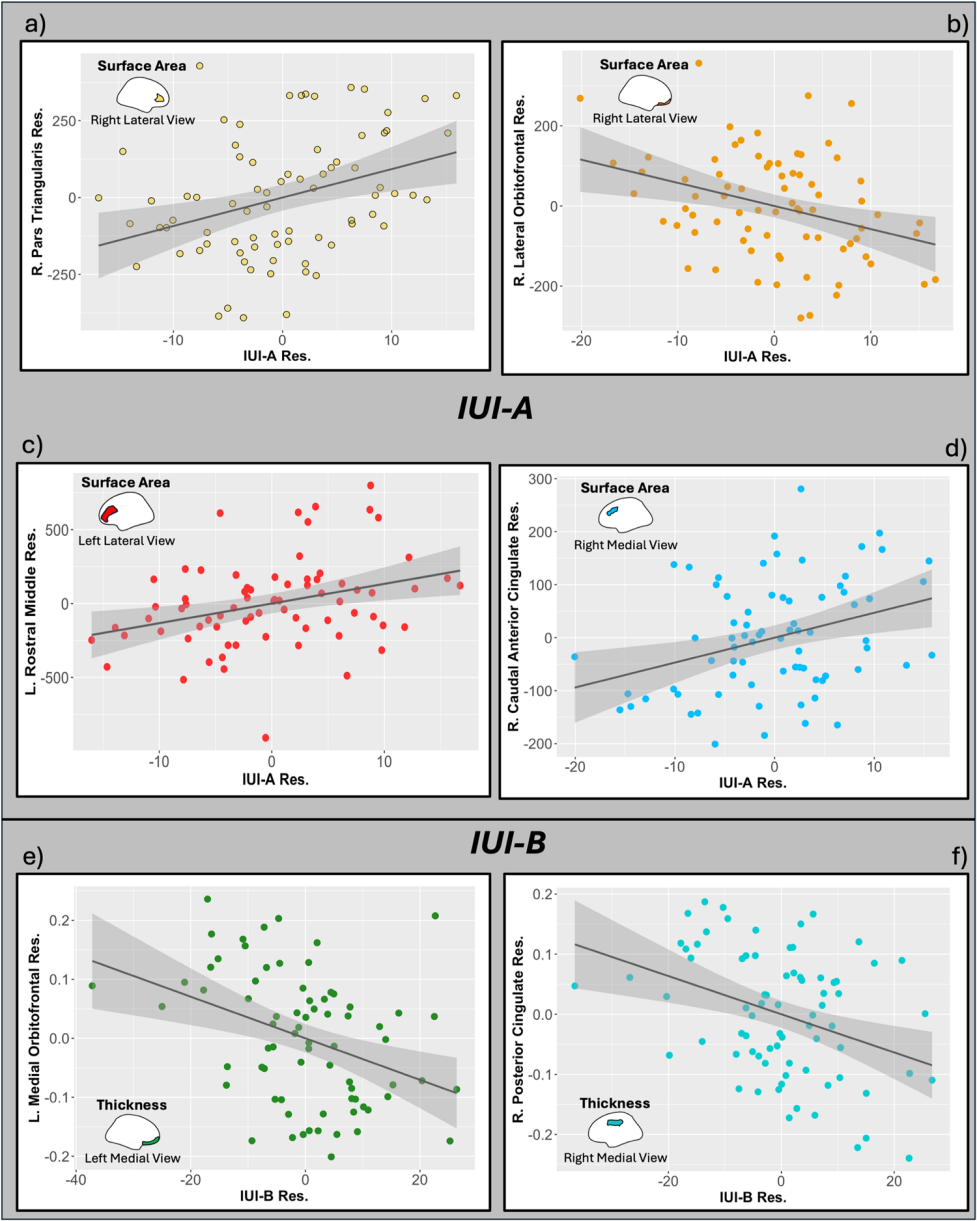
Residual associations in adolescents of IUI-A and IUI-B with characteristics of brain morphology (Top) The associations between the residuals of IUI-A and the residuals of surface area of the (**a**) right pars triangularis, (**b**) right lateral orbitofrontal, (**c**) left rostral middle frontal gyrus, and (**d**) the right caudal anterior cingulate surface area. (Bottom) The associations of IUI-B residuals and the residuals of surface area of (**e**) the left medial orbitofrontal cortex and (**f**) the right posterior cingulate. The grey-shaded regions on the graphs are the 95% confidence intervals. Residuals were calculated controlling for Common Internalizing, Anxious Apprehension, Age, Gender, Gender * Anxious Apprehension, Scanner Code, and Overall Brain Characteristic (e.g., Average Hemispheric Surface Area)

In the presentation of results below, we highlight FDR-corrected significant associations between gray matter morphometry and IUI subscales. As we ran two distinct sets of models (i.e., one set controlling for other aspects of internalizing psychopathology and the other not), for all FDR-significant associations observed in one set, we report the degree to which a similar association was observed in the other set. See Table 3 for a summary of these results. See Figs. 1 and 2 for plots of residuals of IUI and regions of interest in significant models. Complete model outputs for the results that are discussed can be found in Supplementary Tables S4–S7.

**Table 3:** Significant associations between IUI scores and regional gray matter morphometry

Variables			Controlling for psychopathology covariates					Not controlling for psychopathology covariates				
*IUI subscale*	*Measure*	*ROI*	*Estimate*	*Stand. beta*	*t-value*	*unc. p*	*FDR p*	*Estimate*	*Stand. beta*	*t-value*	*unc. p*	*FDR p*
			*(90% CI)*					*(90% CI)*				
***Adults***												
IUI-A	Area	Left pars opercularis	16.67(9.58, 23.76)	.69	4.78	<.001*	<.001^F^	9.99(3.39, 16.58)	.43	3.07	.004*	.098
IUI-A	Volume	Left pallidum	−12.75(−21.24, −4.27)	−.54	−3.06	.004*	.035^F^	−2.54(−9.33, 4.24)	−.12	−0.76	.451	.642
IUI-B	Area	Left pars opercularis	5.50(1.8, 9.19)	.52	3.02	.005*	.038	3.02(−0.61, 6.65)	.25	1.69	.100	.353
IUI-B	Area	Left pars orbitalis	−1.34(−2.24, −0.44)	−.53	−3.04	.005*	.038^F^	−1.31(−2.03, −0.60)	−.49	−3.73	<.001*	.017^F^
IUI-B	Area	Right Insula	−3.90(−6.39, −1.41)	−.48	−3.19	.003*	.038^F^	−2.17(−4.4, 0.06)	−.25	−1.98	.056	.269
***Adolescents***												
IUI-A	Area	Right caudal ant. cingulate	4.70(1.41, 7.98)	.39	2.86	.006*	.041^F^	2.63(0.19, 5.06)	.22	2.15	.035*	.169
IUI-A	Area	Right pars triangularis	9.26(3.16, 15.36)	.45	3.03	.004*	.041^F^	3.28(−1.13, 7.7)	.16	1.48	.143	.489
IUI-A	Area	Right lateral orbitofrontal	−5.76(−9.71, −1.82)	−.23	−2.91	.005*	.041^F^	−4.88(−7.77, −1.99)	−.20	−3.37	.001*	.029^F^
IUI-A	Area	Left rostral middle frontal	12.69(3.62, 21.76)	.20	2.79	.007*	.041^F^	8.31(1.51, 15.11)	.13	2.44	.017*	.139
IUI-B	Thickness	Left medial orbitofrontal	−0.00(−0.01, 0.00)	−.57	−3.22	.002*	.028^F^	−0.00(0.00, 0.00)	−.13	−1.20	.234	.561
IUI-B	Thickness	Right posterior cingulate	−0.00(−0.01, 0)	−.57	−3.17	.002*	.028^F^	−0.00(−0.00, 0.00)	−.07	−0.64	.523	.785

*Note.* results for adults are shown on top portion of the table, and adolescents are shown on the bottom. Results controlling for psychopathology covariates (Common Internalizing Factor Score, Anxious Apprehension-Specific Factor Score) are shown on left. Results not controlling for those factors are shown on right. Measure = gray matter morphometry measure; unc. *p* = uncorrected *p*-value; FDR-*p* = False Discovery Rate corrected *p*-value; IUI-A = Intolerance of Uncertainty Index - subscale A; IUI-B - Intolerance of Uncertainty Index - subscale B

^*^Significant at an uncorrected *p*-value < .05; ^F^Significant at an FDR-corrected *p* < .05; CI = confidence interval; ant. = anterior

### Adults

#### Associations with IUI-A

When including common internalizing and anxious apprehension-specific factor scores as covariates in the adult sample, we observed FDR-corrected significant associations of higher IUI-A with greater surface area of left pars opercularis subregion of the inferior frontal gyrus (std. β = .69, *t* = 4.78, *p* < .001, FDR-*p* < .001), as well as less volume of the left pallidum (std. β = −.542, *t* = −3.06, *p* = .004, FDR-*p* = .035). In models that did not control for common internalizing and anxious apprehension, the association of IUI-A with pars opercularis was significant at an uncorrected level (*p* = .004; FDR-*p* = .098), whereas the association with the pallidum was nonsignificant (*p* = .451; FDR-*p* = .642). No FDR-significant associations were observed with IUI-A in models not controlling for common internalizing and anxious apprehension-specific.

#### Associations with IUI-B

Like IUI-A, when including common internalizing and anxious apprehension-specific factor scores as covariates IUI-B was positively related to the surface area of the left pars opercularis (std. β = .52, *t* = 3.02, *p* = .005, FDR-*p* = .038). When including common internalizing and anxious apprehension-specific factor scores as covariates in the adult sample, after FDR-correction, higher IUI-B scores were associated with less surface area of the left pars orbitalis subregion of the inferior frontal gyrus (std. β = −.53, *t* = −3.04, *p* = .005, FDR-*p* = .038) and the right insula (std. β = −.478, *t* = −3.19, *p* = .003, FDR-*p* = .038). In models that did not control for common internalizing and anxious apprehension, the association of IUI-B with pars orbitalis remained significant at an FDR-corrected level (std. β = −.492, *t* = −3.73, *p* < .001; FDR-*p* = .017), whereas the associations with the right insula and left pars opercularis were marginal at an uncorrected *p*-value (right insula: *p* = .056, FDR-*p* = .269: left pars opercularis: *p* = .100, FDR-*p* = .353). The parallel results in both sets of models for the par orbitalis region of the IFG provides support for a robust relationship with IUI-B.

### Adolescents

#### Associations with IUI-A

When including common internalizing and anxious apprehension-specific factor scores as covariates in the adolescent sample, we observed FDR-corrected significant associations of higher IUI-A with surface area of three right hemisphere ROIs and one in the left hemisphere. Higher IUI-A was associated with greater surface area in the right caudal anterior cingulate (rcACC; std. β = .389, *t* = 2.856, *p* = .006, FDR-*p* = .041) and right pars triangularis subregion of the inferior frontal gyrus (std. β = .451, *t* = 3.03, *p* = .004, FDR-*p* = .041), right lateral orbitofrontal cortex (rlOFC; std. β = −.233, *t* = −2.91, *p* = .005, FDR-*p* = .041), and the left rostral middle frontal gyrus (lrMFGstd. β = .20, *t* = 2.79, *p* = .007, FDR-*p* = .041). In models that did not control for common internalizing and anxious apprehension-specific, the relationship between the rlOFC was significant and passed FDR corrections (rlOFC: *p* = .001, FDR-*p* = .029), whereas for the remaining three regions, they did not (rcACC: *p* = .035, FDR-*p* = .169; lrMFG: *p* = .017; FDR-p= .139; r pars triangularis: *p* = .143; FDR-*p* = .489).

#### Associations with IUI-B

After FDR-correction in models controlling for common internalizing and anxious apprehension-specific, the IUI-B subscale was found to be associated with cortical thickness of the left medial orbitofrontal cortex (lmOFC: std. β = −.570, *t* = −3.215, *p* = .002, FDR-*p* = .028) and right posterior cingulate cortex (rpCC: std. β = −.568, *t* = −3.167, *p* = .002, FDR-*p* = .028). However, neither of these associations were found to be significant in models in which common internalizing and anxious apprehension-specific were not included as covariates (lmOFC: *p* = .234, FDR-*p* = .561; rpCC: *p* = .523, FDR-*p* = .785). No FDR-significant associations were observed with IUI-B in models not controlling for common internalizing and anxious apprehension-specific.

Because of differential changes in brain development and psychopathology in males and females during adolescence (Altemus et al., 2014), we also ran models on each gender separately. Because we had no specific *a priori* hypotheses of gender differences, a presentation of these results is not provided; however, for the interested reader, more details are provided in Supplementary Fig. S3 and Tables S8–11.

## Discussion

The main finding of the present study is that in nonclinical populations associations can be observed between levels of intolerance of uncertainty (IU) and aspects of brain morphology in both middle-aged women as well as adolescents. Furthermore, these associations are independent of any relationship with general symptoms of internalizing disorders (i.e., motivational anhedonia and distress) or specific symptoms related to anxious apprehension (i.e., worry). Perhaps the most notable finding of the current study is that morphological characteristics of multiple subregions of the inferior frontal gyrus (IFG), also referred to as ventrolateral prefrontal cortex (VLPFC), are associated with levels of IU in adult women and adolescents. In adult women, relatively greater surface area of the left pars opercularis is significantly associated with increasing scores on the IUI-A subscale, which measures the degree to which individuals find uncertainty distressing and threatening, and scores on the IUI-B subscale, which measures the consequences of those beliefs. In addition, an association was observed between IUI-B and another portion of the left inferior frontal lobe, the pars orbitalis. In the adolescent sample, levels of IUI-A are also associated with morphology of portions of the inferior frontal lobe, the pars triangularis. However, these effects were observed in the right hemisphere, not the left.

Interestingly, the left inferior frontal gyrus (left VLPFC) is not a region specifically identified in the Grupe & Nitschke (2013) model as playing a role in processes related to intolerance of uncertainty. Rather activation and connectivity of this region has been associated with anxiety, and more specifically anxious apprehension (Engels et al. 2007; Guha et al., 2020), or in common parlance, worry. Yet our analyses indicate an association of intolerance of uncertainty with this region that goes above and beyond any association with common aspects of internalizing symptoms or symptoms more specific to anxious apprehension.

We considered various hypotheses that may provide potential explanations about why there is a specific association between morphology of the left inferior frontal gyrus (VLPFC) and intolerance of uncertainty. While traditionally the left inferior frontal gyrus has been associated with language processing, it has also been hypothesized that portions of this region are involved in more domain-general processing, including cognitive control (Fedorenko & Blank, 2020) and emotional processing (Shiba et al., 2016).

With regards to the cognitive operations enacted by left inferior frontal cortex that might be related to processing ambiguity, we consider three potential possibilities. One hypothesis regarding function of the left inferior frontal cortex is that it is involved in detecting structured sequences and the dependencies between them, whether they be of a linguistic or nonlinguistic nature (see Udden & Bahlmann, 2012, for review). To the degree that someone might have difficulty appreciating the extent and variety of these contingencies or dependences, they might see uncertainty as challenging or threatening. Another viewpoint argues that the left inferior frontal cortex is involved in guiding semantically based aspects of memory retrieval (Badre & Wagner, 2007). To the degree that knowledge stores cannot be used effectively to adjudicate between a variety of possible outcomes, uncertainty may be perceived as problematic.

Finally, our group has shown that the LIFG/VLPFC is specifically involved in aspects of cognitive control that involve selection across task-relevant options, especially when there are numerous plausible responses that could be made (Snyder et al., 2010, 2011). For example, LIFG becomes more activated when there are multiple plausible options in a verb generation task (e.g., generating verbs to the word “ball,” which could include “kick,” “throw,” “catch,” “hit,” etc.) compared with when there is little or no ambiguity with regards to the response (e.g., generating verbs to the word “knife,” in which the predominant response is “cut”). To the degree that selection amongst alternatives might be impaired or altered, dealing with uncertainty would be difficult. Moreover, research has suggested that such LIFG functioning is important for flexible semantic retrieval of thematic and taxonomic relationships amongst information that presumably would be used to construct potential outcomes under situations of ambiguity (Zhang et al., 2021). Finally, connectivity of this region has been linked to individual differences in semantic retrieval abilities (Zhang et al., 2022). We propose that this aspect of cognitive control implemented by the LIFG may undergird the associations of neuroanatomical features of this region with individual differences in levels of intolerance of uncertainty observed in the present study. Regardless of which of these explanations is correct, there exist plausible cognitive mechanisms linking the IFG to intolerance of uncertainty.

With regards to the role that this region may play in emotional processing, evidence from nonhuman animals suggests that VLPFC is particularly important for shifting attention away from salient negative features of information (see Shiba et al., 2016, for review). In humans, these regions are engaged during re-appraisal of negative emotional information to something more positive, which requires shifting focus on item or experiential attributes (“Yes, I just lost my job, but I didn’t like it anyway, and it will allow me to pursue work that is more meaningful.”) (Buhle et al., 2014). To the degree that such shifting is impaired, it might affect that degree to which uncertainty can be tolerated, because uncertainty may require going back and forth to judge the emotional impact of outcomes. It should be noted that left VLPFC has been implicated more generally across a variety of situations in which emotions must be regulated, either amplified or decreased, and across a variety of regulation strategies (Morawetz et al., 2017).

A common thread through both the cognitive and emotional processes supported by VLPFC is that they are related to control or regulatory functions. It should be noted that in the model of Grupe & Nitschke (2013), dorsolateral prefrontal cortex is the brain region that is identified as playing the major role in cognitive control processes with regards to processing of uncertainty. While we observed associations with morphology of this region, they were less robust than for VLFPC. In line with Grupe & Nitschke’s (2013) UAMA model, there is a relationship between surface area of the left rostral middle frontal gyrus (DLPFC) and intolerance of uncertainty in adolescents, and more specifically female adolescents (Fig. S3; Table S8), as well as a trending relationship in our adult sample (see Table S12 for adult model output).

From the perspective of some of our prior theoretical work, the less robust relationship of IU with DLPFC in the current study is potentially understandable. More specifically, we have argued that DLPFC plays a prominent role in actively maintaining task goals, but that left VLPFC selects amongst alternatives that can then be used to support those goals (Snyder et al., 2014). From that perspective, uncertain outcomes can be independent of task goals. For example, one’s goal may be to save up money for retirement, but the uncertainty only arises with regards to how one does so to ensure a suitable nest egg years from now (e.g., save a lot early in life or wait until after major purchases are made, such as a house; purchase an annuity; invest in the stock market, etc.). Said differently, the goal will not vary, but the means to achieving it will and those are likely to involve selection amongst uncertain outcomes.

While there are a variety of theories with regards to the role of the right inferior frontal cortex, which is the region of VLPFC for which an association was observed with IUI-A in adolescents, all argue that it plays some role in cognitive control. One prominent model suggests that it is particularly important in inhibitory processes (Aron et al., 2004, 2014), which one might imagine could be important in selecting or suppressing certain choices under conditions of uncertainty. Other viewpoints argue that this region is important for determining what information present in the environment should help to guide goal-oriented behavior (Chatham et al., 2012; Hampshire et al., 2010).

This latter perspective may potentially provide some insight as to the distinct lateralized effects observed for adolescents as compared to adults, with associations of intolerance of uncertainty observed for right inferior frontal regions in adolescents, but with left inferior frontal regions in adults. We speculate that perhaps because adolescents have less experience in life than adults, the selection of relevant information in the environment may play a somewhat important role in how well they evaluate (and tolerate) uncertainty. In contrast, for adults, who have more life experience, left inferior regions, which are involved in selecting amongst goal-related alternatives actions, may be more important in determining how well they evaluate (and plan for) uncertainty.

The morphology of two other regions, portions of the orbitofrontal cortex, and portions of the cingulate, showed significant associations with intolerance of uncertainty in only the adolescent sample. In the adolescents, associations were observed with right lateral and left medial orbitofrontal cortex. Although there are different viewpoints on the exact operations that are undergirded by orbitofrontal cortex, most viewpoints across animal and human studies assume that it plays a role in predicting the outcomes associated with future events (Shiba et al., 2016), especially those that have not been directly experienced but can only be inferred (Kahnt & Schoenbaum, 2021). In this sense, it may be that orbitofrontal cortex plays a role in making inferences about uncertain outcomes that may compliment semantic retrieval guided by VLPFC that searches through semantic stores.

With regards to the cingulate, associations in medial anterior sections of the cingulate, known as the midcingulate cortex, were observed in adolescents. The caudal anterior cingulate region has been implicated in attentional orientation to assess the self-relevance of objects and events (Vogt, 2019), which would help individuals determine what elements of a situation or decision are most important to them under conditions of uncertainty. In contrast, the midcingulate cortex has been proposed to be involved in selection of actions based on the amount of reward compared with pain or aversive properties of a movement (Vogt, 2016), which would need to be calculated in uncertain outcomes. It should be noted that while we performed an exploratory direct contrast of the cingulate effects between adolescents and adults, it did not yield significant results. Nonetheless, the pattern we observed in these findings are consistent with those of Krain et al. (2006, 2008), suggesting differentiation in the portions of the anterior cingulate related to intolerance of uncertainty in adolescents compared with adults. Taken together, these results implicate morphological characteristics of the inferior and anterior regions of the lateral and medial prefrontal cortex as being implicated in individual differences in intolerance of uncertainty.

## Limitations

While this study revealed specific associations, it is not without limitations. First, our adult sample was restricted to women. The degree to which similar findings would be found in adult males remains to be seen, although we are not aware of any reports of sex differences with regards to intolerance of uncertainty. Second, it is difficult to know the degree to which these differences in morphology actually influence the functioning of these regions. Third, our results were observed across both surface area and thickness with no specific discernable pattern. In some ways, this outcome is not surprising, because currently there is no consensus regarding which of these two aspects of brain morphology are likely to be associated with individual differences, although they are known to be driven by different developmental process and genetic influences (Im et al., 2008; Panizzon et al., 2009). More specifically, it is thought that surface area is influenced by neuronal migration during the early development of the cortex, whereas cortical thickness is influenced by processes of neuronal proliferation and pruning (Rakic, 1988). Fourth, we cannot draw clear conclusions on whether there are developmental differences in the association between Intolerance of Uncertainty and brain morphology. While some differences were observed in the pattern of associations between our adult and adolescent samples, a direct comparison did not yield significant results, making conclusions on this score indeterminate. Fifth, while the IUI scale differentiates between cognitions and attitudes regarding intolerance of uncertainty (IUI-A) and the way those attitudes manifest (e.g., worry) and are acted on (e.g., avoidance of situations involving uncertainty) (IUI-B), we did not see a clear differentiation in the pattern of association with each of these scales. Hence, it is likely prudent to be cautious in making any strong conclusions based on the scale for which an association was observed. Sixth, our sample sizes were relatively small (N~40, N~80) for individual differences research. Nonetheless, our investigation is likely to be more highly powered than typical studies with these sample sizes that only obtain data at one timepoint. By averaging both behavioral and neuroanatomical data over two timepoints separated by 2 years, we are much more likely to be measuring stable aspects of individual differences than effects that might be associated with data acquisition at a single timepoint (Smith et al., 2023; Baker et al., 2021).

## Conclusions

The current study provides evidence that aspects of brain morphology in inferior lateral and medial portions of the prefrontal cortex are specifically associated with individual differences in intolerance of uncertainty, above and beyond any associations with other symptoms of internalizing psychology (e.g., motivational anhedonia and distress, worry). This pattern was observed both for a sample of adult women and for a mixed gender sample of adolescents, suggesting that these associations may span different developmental time periods. The brain regions implicated in the current study are those involved in a variety of cognitive and emotional processes, ranging from the selection of information when the means of doing so is underspecified, calculating the relative outcome of events based both on inference and on prior knowledge, and selection of actions that may lead to rewarding or punishing outcomes. These findings are consistent with prior theoretical models that suggest intolerance of uncertainty is influenced by processing in a wide variety of brain regions (Grupe & Nitschke, 2013).

## Supplementary information

Below is the link to the electronic supplementary material.

Supplementary file1 (DOCX 678 KB)

## Data Availability

All the data for the analyses reported in the current study, along with the code for the analytic pipeline for the analyses reported in this paper can be found at https://osf.io/bmp9r/.
