# Correction: Individual differences in intolerance of uncertainty is primarily linked to the structure of inferior frontal regions

**DOI:** 10.3758/s13415-025-01288-y

**Published:** 2025-03-18

**Authors:** Kenneth W. Carlson, Harry R. Smolker, Louisa L. Smith, Hannah R. Snyder, Benjamin L. Hankin, Marie T. Banich

**Affiliations:** 1 Institute of Cognitive Science, University of Colorado Boulder, Boulder, CO USA; 2 Department of Psychology & Neuroscience, University of Colorado Boulder, D447C Muenzinger Hall, UCB 345, Boulder, CO 80309 USA; 3 Department of Psychology, Brandeis University, Waltham, MA USA; 4 Department of Psychology, University of Illinois at Urbana-Champaign, Champaign, IL USA

**Correction to: Cognitive, Affective, & Behavioral Neuroscience**

10.3758/s13415-024-01262-0

The original article has been updated to correct author ‘s name: Hannah R. Snyder. It was published incorrectly as Hannah R. Synder.

