# Supplementary material for: Individual differences in intolerance of uncertainty is primarily linked to the structure of inferior frontal regions
Source: Cogn Affect Behav Neurosci. 2025 Jan 27;25(3):727–43. doi: 10.3758/s13415-024-01262-0 (PMC12129862; doi:10.3758/s13415-024-01262-0)
Supplement: Supplementary file 1 — Supplementary file1 (DOCX 678 KB) [file 13415_2024_1262_MOESM1_ESM.docx]

**SUPPLEMENTARY MATERIALS**

S1. Additional Information on the Derivation of Factor Scores and Model Fit for Factors Related to Psychopathology

1. *Overview of the overall factor analysis*

Using responses to the MASQ and PWSQ, we applied the bifactor model discussed in Snyder et al., 2022 to the adolescent and adult samples using confirmatory factor analyses to derive factor scores for levels of Common internalizing symptoms as well as Anxious Apprehension-specific symptoms, Anxious-Arousal-specific symptoms and Low Positive Affect-specific symptoms. Only the Common Internalizing factor scores and Anxious Apprehension-specific factor scores are of interest for the current report.

Factor scores were computed separately for each sample and at each timepoint, resulting in a total of four sets of factor scores. For the adult sample, factor scores at timepoint 1 were computed across a larger sample which also included 117 individuals recruited from University of Illinois, Urbana-Champaign, whereas at timepoint 2 factor scores were computed within the current adult sample, only. Adolescent factor scores were computed using participants from the current study only at both timepoints. Sample sizes and model fit indices are reported below.

Given the relatively large number of indicators and small sample size, an approach of correlational parceling of related indicators was used (Little et al., 2013), first two items with the highest correlations were averaged and then highly correlated two-item parcels were averaged together to create four-item parcels. For scales with an odd number of items, the single item was averaged with the parcel in which they were most highly correlated with. In doing so, we were able to simultaneously reduce the number of indicators and preserve the construct structure. The current project focuses on the Common Internalizing and Anxious Apprehension-Specific factors.

1. *Models for Adults*

Including the supplementary sample from the Urbana-Champaign, Illinois area, the timepoint one adult sample consisted of 204 individuals while the timepoint 2 sample consisted of 177 individuals. Confirmatory factor analysis suggested that the bifactor model provided a This newly revised model had good to acceptable fit for the timepoint one sample (CFI = 0.95, RMSEA = 0.067, SRMR = 0.070; χ2 (90) = 172.28, *p* < .001), and all parcels loaded significantly on their factor(s). Factor scores were then saved for further analysis. Factor determinacies (a measure of the degree to which factor scores represent the true latent factors, > 0.9 is ideal) were high for the Common Internalizing (CI) (0.91), Low Positive Affect (LPA)-specific (0.91), and Anxious Apprehension-specific (AAp) (0.92) factors, but lower for the Anxious Arousal-specific (AAr) factor (0.65). The model for timepoint 2 also had good to acceptable fit (CFI=.95, RMSEA=.064, SRMR=.061; χ2 (91) = 157.72, p<.001). Factor determinacies for the timepoint 2 model were, Common Internalizing (0.91), LPA-specific (0.89), AAp (0.93) and AAr (0.74).

*c. Model for Adolescents:*

The adolescent sample consisted of 188 individuals at Timepoint 1 and 98 at Timepoint 2. Model fit for Timepoint 1 was good (CFI = .966, RMSEA = .056, SRMR = .072; χ2(92) = 130.958, *p* < .005). Model determinacies were Common Internalizing factor (.90), Low Positive Affect-specific factor (.93), and Anxious Apprehension-specific factor (.93), Anxious Arousal-specific factor (.87). For timepoint 2 model fit was once again good (CFI=.970, RMSEA=.056, SRMR=.056; χ2(89) = 116.20, p = .028). Model determinacies were common internalizing factor (.91), Low Positive Affect -specific factor (.92), and Anxious Apprehension-specific factor (.91), Anxious Arousal-specific factor (.78).

**S2.** Adolescent Gender Specific Results

Models controlling for common internalizing and anxious apprehension-specific factor scores revealed three significant relationships that were not observed for the group as a whole. In female adolescents, greater IUI-A scores were found to be associated with greater surface area of the right rostral middle frontal (std. β=.305, *t*= 3.14, FDR-*p*= .030) and greater surface are of the right medial orbitofrontal (std. β= .421, *t*= 3.49, FDR-*p*= .023) (Table S8). In the male sample higher IUI-B was associated with less surface area of the left rostral anterior cingulate (std. β= -.69, *t*= -4.00, FDR-*p*= .010)(Table S10). In models where IUI-A or B are the only predictors of interest in the model we find associations with IUI-A continue to be significant. In the female sample we found that greater IUI-A was associated with greater surface area of two regions, the right hemisphere rostral middle frontal gyrus (std. β= .30, t= 3.92, fdr-p= .005) and the right medial orbitofrontal cortex (std. β= .46, t= 4.89, fdr-p< .001)(Table S9). The relationship between IUI-B and the left rostral anterior cingulate in the male only sample was not significant when internalizing symptoms were not accounted for (fdr-p=.778)(Table S11).

Figure S1


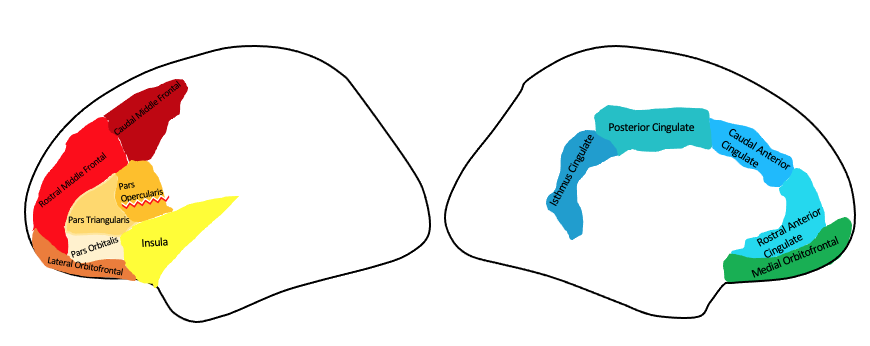


Figure S2


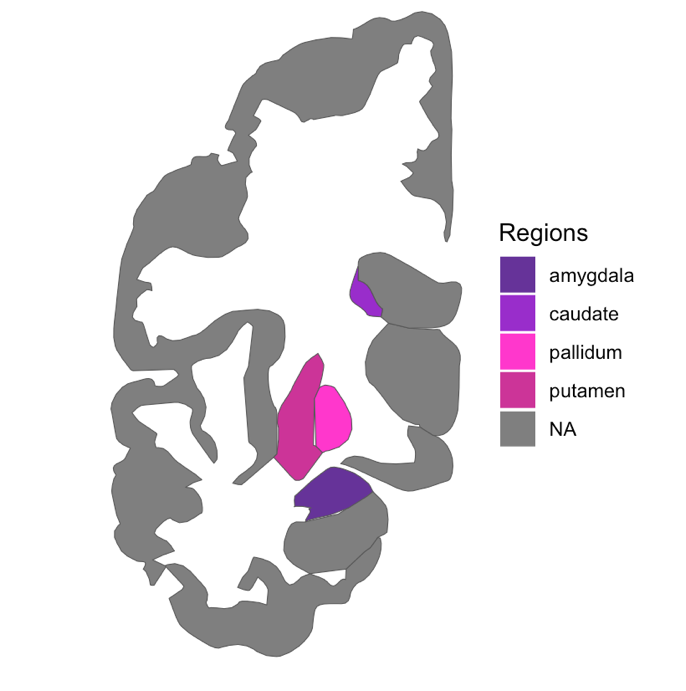


Table S1

| **Adults** |  |  |  |  |  |  | |  | |
| --- | --- | --- | --- | --- | --- | --- | --- | --- | --- |
| Measures | 1a | 1b | 2a | 2b | 3a | 3b | | 4a | |
| 1a. IUI-A TP1 |  |  |  |  |  |  | |  | |
| 1b. IUI-A TP2 | 0.67**** |  |  |  |  |  | |  | |
| 2a. IUI-B TP1 | 0.78**** | 0.61**** |  |  |  |  | |  | |
| 2b. IUI-B TP2 | 0.62**** | 0.75**** | 0.73**** |  |  |  | |  | |
| 3a. AAp TP1 | 0.46** | 0.44** | 0.53*** | 0.36* |  |  | |  | |
| 3b. AAp TP2 | 0.51*** | 0.49** | 0.51*** | 0.45** | 0.74**** |  | |  | |
| 4a. Comm. Int. TP1 | 0.22 | 0.19 | 0.44** | 0.30• | 0.16 | 0.16 | |  | |
| 4b. Comm. Int. TP2 | 0.2 | 0.33* | 0.32* | 0.33* | 0.15 | 0.15 | | 0.64**** | |
| **Adolescents** | | |  |  |  |  |  | |  |
| Measures | 1a | 1b | 2a | 2b | 3a | 3b | | 4a | |
| 1a. IUI-A TP1 |  |  |  |  |  |  | |  | |
| 1b. IUI-A TP2 | 0.46**** |  |  |  |  |  | |  | |
| 2a. IUl-B TP1 | 0.86**** | 0.40*** |  |  |  |  | |  | |
| 2b. IUI-B TP2 | 0.45**** | 0.85**** | 0.47**** |  |  |  | |  | |
| 3a. AAp TP1 | 0.50**** | 0.23* | 0.50**** | 0.32** |  |  | |  | |
| 3b. AAp TP2 | 0.25* | 0.44**** | 0.25* | 0.53**** | 0.47**** |  | |  | |
| 4a. Comm. Int. TP1 | 0.57**** | 0.26* | 0.62**** | 0.35** | 0.15 | 0.07 | |  | |
| 4b. Comm. Int. TP2 | 0.46**** | 0.49**** | 0.49**** | 0.62**** | 0.29* | 0.13 | | 0.52**** | |

Table S2

| **Adults** |  | | | | | | | | |  |
| --- | --- | --- | --- | --- | --- | --- | --- | --- | --- | --- |
| Measures | 1a | 1b | 2a | 2b | 3a | 3b | 4a | 4b | 5a | |
| 1a. Left MSA TP1 |  |  |  |  |  |  |  |  |  | |
| 1b. Left MSA TP2 | 0.98**** |  |  |  |  |  |  |  |  | |
| 2a. Right MSA TP1 | 0.99**** | 0.98**** |  |  |  |  |  |  |  | |
| 2b. Right MSA TP2 | 0.97**** | 0.99**** | 0.98**** |  |  |  |  |  |  | |
| 3a. Left MT TP1 | 0.1 | 0.1 | 0.09 | 0.11 |  |  |  |  |  | |
| 3b. Left MT TP2 | 0.13 | 0.09 | 0.11 | 0.08 | 0.85**** |  |  |  |  | |
| 4a. Right MT TP1 | 0.01 | 0.03 | 0 | 0.02 | 0.96**** | 0.85**** |  |  |  | |
| 4b. Right MT TP1 | 0.07 | 0 | 0.04 | -0.02 | 0.77**** | 0.93**** | 0.80**** |  |  | |
| 5a. EIV TP1 | 0.63**** | 0.61**** | 0.59**** | 0.57**** | 0.16 | 0.22 | 0.12 | 0.19 |  | |
| 5b. EIV TP2 | 0.55*** | 0.52*** | 0.51*** | 0.48** | 0.15 | 0.22 | 0.09 | 0.22 | 0.89**** | |
| **Adolescents** | | |  |  |  |  |  |  |  | |
| Measures | 1a | 1b | 2a | 2b | 3a | 3b | 4a | 4b | 5a | |
| 1a. Left MSA TP1 |  |  |  |  |  |  |  |  |  | |
| 1b. Left MSA TP2 | 0.99**** |  |  |  |  |  |  |  |  | |
| 2a. Right MSA TP1 | 0.99**** | 0.98**** |  |  |  |  |  |  |  | |
| 2b. Right MSA TP2 | 0.99**** | 0.99**** | 0.99**** |  |  |  |  |  |  | |
| 3a. Left MT TP1 | -0.14 | -0.18 | -0.12 | -0.15 |  |  |  |  |  | |
| 3b. Left MT TP2 | -0.05 | -0.08 | -0.03 | -0.06 | 0.82**** |  |  |  |  | |
| 4a. Right MT TP1 | -0.12 | -0.15 | -0.13 | -0.14 | 0.93**** | 0.78**** |  |  |  | |
| 4b. Right MT TP1 | -0.02 | -0.04 | -0.01 | -0.03 | 0.82**** | 0.94**** | 0.83**** |  |  | |
| 5a. EIV TP1 | 0.90**** | 0.89**** | 0.90**** | 0.89**** | 0.04 | 0.11 | 0.06 | 0.13 |  | |
| 5b. EIV TP2 | 0.89**** | 0.88**** | 0.88**** | 0.88**** | 0.05 | 0.11 | 0.06 | 0.11 | 0.97**** | |

Table S3

| **Adults** |  |  |  |  |  |  |  |  |
| --- | --- | --- | --- | --- | --- | --- | --- | --- |
| Measures | 1 | 2 | 3 | 4 | 5 | 6 | 7 | 8 |
| 1. Avg. IUI-A |  |  |  |  |  |  |  |  |
| 2. Avg. IUI-B | 0.81**** |  |  |  |  |  |  |  |
| 3. Avg. AAp | 0.56*** | 0.53*** |  |  |  |  |  |  |
| 4. Avg. Comm. Int. | 0.29• | 0.40** | 0.18 |  |  |  |  |  |
| 5. Avg. EIV | -0.27• | -0.34* | -0.01 | 0.12 |  |  |  |  |
| 6. Avg. Left MSA | -0.16 | -0.21 | -0.07 | -0.03 | 0.59**** |  |  |  |
| 7. Avg. Right MSA | -0.12 | -0.18 | -0.04 | -0.05 | 0.55*** | 0.99**** |  |  |
| 8. Avg. Left MT | -0.13 | -0.24 | -0.15 | -0.2 | 0.2 | 0.11 | 0.1 |  |
| 9. Avg. Right MT | -0.08 | -0.18 | -0.14 | -0.21 | 0.16 | 0.03 | 0.01 | 0.96**** |
| **Adolescents** |  |  |  |  |  |  |  |  |
| Measures | 1 | 2 | 3 | 4 | 5 | 6 | 7 | 8 |
| 1. Avg. IUI-A |  |  |  |  |  |  |  |  |
| 2. Avg. IUI-B | 0.88**** |  |  |  |  |  |  |  |
| 3. Avg. AAp | 0.49**** | 0.55**** |  |  |  |  |  |  |
| 4. Avg. Comm. Int. | 0.61**** | 0.69**** | 0.23* |  |  |  |  |  |
| 5. Avg. EIV | -0.11 | -0.12 | -0.31** | 0.05 |  |  |  |  |
| 6. Avg. Left MSA | -0.12 | -0.13 | -0.23* | 0.01 | 0.90**** |  |  |  |
| 7. Avg. Right MSA | -0.13 | -0.15 | -0.24* | 0.01 | 0.90**** | 0.99**** |  |  |
| 8. Avg. Left MT | -0.14 | -0.05 | -0.13 | 0.07 | 0.08 | -0.12 | -0.1 |  |
| 9. Avg. Right MT | -0.14 | -0.06 | -0.15 | 0.02 | 0.09 | -0.09 | -0.08 | 0.95**** |

**ADULT SAMPLE**

Table S4

| IUI - A | | | | | | | | | |
| --- | --- | --- | --- | --- | --- | --- | --- | --- | --- |
| ROI | N | r2/  r2 adj. | IV | Est. | Std. Est. | Conf. Int. | t-value | P | FDR-P |
| Left Pars Opercularis  Surface Area | 40 | 0.573/  0.496 | IUI-A | 16.7 | 0.687 | 9.58, 23.8 | 4.78 | **<.001** | **<.001** |
|  |  |  | Com. Int. | -182 | -0.217 | -385, 21.7 | -1.82 | 0.078 | 0.269 |
|  |  |  | AAp | -185 | -0.505 | -285, -83.8 | -3.72 | **<.001** | **0.017** |
|  |  |  | Left MSA | 0.020 | 0.520 | 0.01, 0.03 | 4.39 | **<.001** | **<.001** |
|  |  |  | Age (Months) | 0.304 | 0.115 | -0.33, 0.93 | 0.983 | 0.333 | 0.595 |
|  |  |  | Scanner Code | -117 | -0.219 | -244, 11.2 | -1.86 | 0.072 | 0.424 |
|  |  |  | (Intercept) | -544 | NA | -1504, 417 | -1.15 | 0.258 | 0.563 |
| Left Pallidum  Volume | 40 | 0.401/  0.292 | IUI-A | -12.8 | -0.542 | -21.2, -4.27 | -3.06 | **<.001** | **0.035** |
|  |  |  | Com. Int. | 246 | 0.318 | 11.8, 481 | 2.14 | **0.040** | 0.160 |
|  |  |  | AAp | 119 | 0.335 | 3.43, 235 | 2.09 | **0.044** | 0.351 |
|  |  |  | EIV | <.001 | -0.010 | 0.00, 0.00 | -0.068 | 0.946 | 0.946 |
|  |  |  | Age (Months) | -0.719 | -0.297 | -1.38, -0.05 | -2.20 | **0.035** | 0.07 |
|  |  |  | Scanner Code | 124 | 0.263 | -7.58, 255 | 1.92 | 0.064 | 0.256 |
|  |  |  | (Intercept) | 1970 | NA | 994, 2950 | 4.10 | **<.001** | **0.001** |
| IUI-B | | | | | | | | | |
| Left Pars Opercularis Surface Area | 40 | 0.408/  0.300 | IUI-B | 5.49 | 0.520 | 1.8, 9.19 | 3.02 | **0.005** | **0.038** |
|  |  |  | Com. Int | -107 | -0.115 | -390, 176 | -0.770 | 0.447 | 0.631 |
|  |  |  | AAp | -156 | -0.438 | -273, -39.7 | -2.73 | **0.010** | 0.229 |
|  |  |  | Left MSA | 0.020 | 0.520 | 0.01, 0.03 | 3.59 | **0.001** | **0.002** |
|  |  |  | Age (Months) | 0.094 | 0.034 | -0.71, 0.9 | 0.236 | 0.815 | 0.850 |
|  |  |  | Scanner Code | -61.2 | -0.123 | -199, - 76.9 | -0.902 | 0.374 | 0.598 |
|  |  |  | (Intercept) | -319 | NA | -1554, 917 | -0.524 | 0.604 | 0.905 |
| Left Pars Orbitalis Surface Area | 39 | 0.441/  0.336 | IUI-B | -1.34 | -0.533 | -2.24, -0.44 | -3.04 | **0.005** | **0.038** |
|  |  |  | Comm. Int. | 68.4 | 0.336 | 6.59, 130.13 | 2.25 | **0.031** | 0.187 |
|  |  |  | AAp | 1.88 | 0.022 | -26.14, 29.91 | 0.137 | 0.892 | 0.926 |
|  |  |  | Left MSA | 0.002 | 0.265 | 0.00, 0.01 | 1.91 | 0.065 | 0.071 |
|  |  |  | Age (Months) | -0.133 | -0.210 | -0.31, 0.04 | -1.54 | 0.133 | 0.637 |
|  |  |  | Scanner Code | -0.316 | -0.003 | -34.4, 33.8 | -0.019 | 0.985 | 0.999 |
|  |  |  | (Intercept) | 563 | NA | 296, 829 | 4.30 | **<.001** | **<.001** |
| Right Insula  Surface Area | 40 | 0.592/0.517 | IUI-B | -3.90 | -0.48 | -6.39, 1.41 | -3.19 | **0.003** | **0.038** |
|  |  |  | Comm. Int. | 206 | 0.34 | 47.6, 365 | 2.64 | **0.013** | 0.1 |
|  |  |  | AAp | -6.79 | -0.025 | -77.7, 64.1 | -0.195 | 0.847 | 0.926 |
|  |  |  | Right MSA | 0.012 | 0.43 | 0.01, 0.02 | 3.61 | **0.001** | **0.002** |
|  |  |  | Age (Months) | 0.097 | 0.05 | -0.34, 0.53 | 0.455 | 0.652 | 0.767 |
|  |  |  | Scanner Code | -106 | -0.29 | -192, 21.2 | -2.54 | **0.016** | 0.127 |
|  |  |  | (Intercept) | 1446 | NA | 737, 2156 | 4.15 | **<.001** | **<.001** |

Table S5

| IUI - A | | | | | | | | | |
| --- | --- | --- | --- | --- | --- | --- | --- | --- | --- |
| ROI | N | r2/  r2 adj. | IV | Est. | Std. Est. | Conf. Int. | t-value | P | FDR-P |
| Left Pars Opercularis  Surface Area | 40 | 0.333/0.257 | IUI-A | 9.99 | 0.430 | 3.39, 16.6 | 3.07 | **0.004** | 0.098 |
|  |  |  | Left MSA | 0.018 | 0.413 | 0.00, 0.03 | 2.76 | **0.009** | **0.011** |
|  |  |  | Age (Months) | -0.066 | -0.025 | -0.81, 0.68 | -0.18 | 0.86 | 0.860 |
|  |  |  | Scanner Code | -42.2 | -0.081 | -197, 113 | -0.55 | 0.584 | 0.888 |
|  |  |  | (Intercept) | 12.5 | NA | -1276, 1301 | 0.020 | 0.984 | 0.984 |
| Left Pallidum  Volume | 40 | 0.148/0.051 | IUI-A | -2.54 | -0.125 | -9.33, 4.24 | -0.76 | 0.451 | 0.642 |
|  |  |  | EIV | <.001 | 0.09 | 0.00, 0.00 | 0.56 | 0.580 | 0.580 |
|  |  |  | Age (Months) | -0.576 | -0.229 | -1.39, 0.23 | -1.44 | 0.158 | 0.205 |
|  |  |  | Scanner Code | 108 | 0.244 | -36.3, 252 | 1.52 | 0.138 | 0.359 |
|  |  |  | (Intercept) | 1337 | NA | 364, 2312 | 2.79 | **0.009** | **0.017** |
|  | | | | | | | | | |
| IUI-B | | | | | | | | | |
| ROI | N | r2/  r2 adj. | IV | Est. | Std. Est. | Conf. Int. | t-value | P | FDR-P |
| Left Pars Opercularis Surface Area | 39 | 0.259/0.172 | IUI-A | 3.019 | 0.252 | -0.61, 6.65 | 1.69 | 0.099 | 0.353 |
|  |  |  | Left MSA | 0.019 | 0.411 | 0.00, 0.03 | 2.68 | **0.011** | **0.012** |
|  |  |  | Age (Months) | -0.056 | -0.021 | -0.86, 0.75 | -0.14 | 0.889 | 0.889 |
|  |  |  | Scanner Code | -58.1 | -0.117 | -213, 96.2 | -0.77 | 0.449 | 0.687 |
|  |  |  | (Intercept) | 59.4 | NA | -1291, 1410 | 0.089 | 0.929 | 0.932 |
| Left Pars Orbitalis Surface Area | 38 | 0.447/0.38 | IUI-A | -1.31 | -0.4918 | -2.03, -0.6 | -3.73 | **<.001** | **0.017** |
|  |  |  | Left MSA | 0.004 | 0.3791 | 0.00, 0.01 | 2.72 | **0.010** | **0.012** |
|  |  |  | Age (Months) | -0.0654 | -0.096 | -0.26, 0.13 | -0.69 | 0.495 | 0.709 |
|  |  |  | Scanner Code | 11.844 | 0.0942 | -22.1, 45.8 | 0.71 | 0.482 | 0.687 |
|  |  |  | (Intercept) | 388.5 | NA | 93.2, 684 | 2.68 | **0.012** | 0.069 |
| Right Insula  Surface Area | 39 | 0.498/0.439 | IUI-A | -2.17 | -0.252 | -4.4. 0.06 | -1.98 | 0.056 | 0.353 |
|  |  |  | Right MSA | 0.014 | 0.492 | 0.01, 0.02 | 3.72 | **<.001** | **0.001** |
|  |  |  | Age (Months) | 0.118 | 0.064 | -0.35, 0.59 | 0.51 | 0.614 | 0.709 |
|  |  |  | Scanner Code | -96.0 | -0.265 | -188, -4.42 | -2.13 | **0.041** | 0.282 |
|  |  |  | (Intercept) | 1163 | NA | 390, 1938 | 3.06 | **0.004** | **0.035** |

**ADOLESCENT SAMPLE**

Table S6

| IUI - A | | | | | | | | | |
| --- | --- | --- | --- | --- | --- | --- | --- | --- | --- |
| ROI | N | r2/  r2 adj. | IV | Est. | Std. Est. | Conf. Int. | t-value | P | FDR-P |
| Right Caudal Anterior Cingulate Surface Area | 74 | 0.448/  0.38 | IUI-A | 4.70 | 0.389 | 1.41, 7.98 | 2.86 | **0.006** | **0.041** |
|  |  |  | Comm. Int. | -85.8 | -0.273 | -162, -9.48 | -2.25 | **0.028** | 0.277 |
|  |  |  | AAp | -3.04 | -0.013 | -72.4, 66.3 | -0.088 | 0.931 | 0.988 |
|  |  |  | Gender | -0.403 | -0.001 | -73.4, 72.6 | -0.011 | 0.991 | 0.991 |
|  |  |  | Right MSA | 0.011 | 0.672 | 0.01, 0.01 | 5.56 | **<.001** | **<.001** |
|  |  |  | Age (Months) | 0.301 | 0.040 | -1.22, 1.82 | 0.396 | 0.693 | 0.844 |
|  |  |  | Scanner Code | 15.7 | 0.048 | -47.7, 79.1 | 0.494 | 0.623 | 0.872 |
|  |  |  | Worry x Gender | 26.7 | 0.064 | -78.7, 132 | 0.506 | 0.614 | 0.819 |
|  |  |  | (Intercept) | -377.2 | NA | -940, 185 | -1.340 | 0.185 | 0.370 |
| Right Pars Triangularis Surface Area | 77 | 0.333/  0.254 | IUI-A | 9.26 | 0.451 | 3.16, 15.4 | 3.03 | **0.004** | **0.041** |
|  |  |  | Comm. Int. | -194 | -0.36 | -333, -54.3 | -2.77 | **0.007** | 0.170 |
|  |  |  | AAp | -1.03 | -0.003 | -140, 138 | -0.02 | 0.988 | 0.988 |
|  |  |  | Gender | -88.5 | -0.195 | -219, 42.3 | -1.35 | 0.181 | 0.744 |
|  |  |  | Right MSA | 0.007 | 0.258 | 0.00, 0.01 | 1.98 | 0.052 | 0.052 |
|  |  |  | Age (Months) | -1.52 | -0.118 | -4.26, 1.21 | -1.11 | 0.270 | 0.719 |
|  |  |  | Scanner Code | -58.9 | -0.100 | -178, 60.5 | -0.98 | 0.328 | 0.818 |
|  |  |  | Worry x Gender | -150 | -0.218 | -341, 41.5 | -1.56 | 0.123 | 0.354 |
|  |  |  | (Intercept) | 1138 | NA | 126, 2150 | 2.24 | **0.028** | 0.169 |
| Right Lateral Orbitofro-ntal Surface Area | 76 | 0.785/  0.76 | IUI-A | -5.80 | -0.233 | -9.71, -1.82 | -2.91 | **0.005** | **0.041** |
|  |  |  | Comm. Int. | 50.9 | 0.081 | -40.3, 142 | 1.11 | 0.269 | 0.808 |
|  |  |  | AAp | 47.2 | 0.097 | -39.3, 134 | 1.09 | 0.280 | 0.564 |
|  |  |  | Gender | -16.7 | -0.031 | -104, 70.5 | -0.382 | 0.704 | 0.982 |
|  |  |  | Right MSA | 0.028 | 0.833 | 0.02, 0.03 | 11.10 | **<.001** | **<.001** |
|  |  |  | Age (Months) | -0.22 | -0.015 | -2.06, 1.61 | -0.24 | 0.808 | 0.844 |
|  |  |  | Scanner Code | 115.4 | 0.177 | 38.7, 192 | 3.01 | **0.004** | **0.044** |
|  |  |  | Worry x Gender | -93.52 | -0.116 | -221, 33.5 | -1.50 | 0.146 | 0.354 |
|  |  |  | (Intercept) | 319.1 | NA | -386, 1024 | 0.904 | 0.369 | 0.653 |
| Left Rostral Middle Frontal Surface Area | 73 | 0.844/  0.824 | IUI-A | 12.7 | 0.20 | 3.62 - 21.76 | 2.79 | **0.007** | **0.041** |
|  |  |  | Comm.Int. | -146 | -0.09 | -352, 59.8 | -1.42 | 0.161 | 0.63 |
|  |  |  | AAp | -82.2 | -0.07 | -292, 128 | -0.78 | 0.437 | 0.74 |
|  |  |  | Gender | -106 | -0.08 | -300, 88.1 | -1.09 | 0.279 | 0.74 |
|  |  |  | Left MSA | 0.07 | 0.88 | 0.06, 0.08 | 13.7 | **<.001** | **<.001** |
|  |  |  | Age (Months) | 0.82 | 0.02 | -3.6, 5.24 | 0.37 | 0.712 | 0.84 |
|  |  |  | Scanner Code | 209 | 0.13 | 37.8, 381 | 2.44 | **0.018** | 0.14 |
|  |  |  | Worry x Gender | 430 | 0.20 | 134, 725 | 2.90 | **0.005** | 0.12 |
|  |  |  | (Intercept) | -1071 | NA | -2615, 473 | -1.39 | 0.171 | 0.37 |
|  | | | | | | | | | |
| IUI – B | | | | | | | | | |
| ROI | N | r2/  r2 adj. | IV | Est. | Std. Est. | Conf. Int. | t-value | P | FDR-P |
| Left Medial Orbitofro-ntal Thickness | 73 | 0.353/  0.272 | IUI-B | -0.004 | -0.57 | -0.01, 0 | -3.22 | **0.002** | **0.028** |
|  |  |  | Comm. Int. | 0.17 | 0.546 | 0.07, 0.27 | 3.45 | **0.001** | **0.024** |
|  |  |  | AAp | 0.032 | 0.139 | -0.04, 0.11 | 0.84 | 0.406 | 0.702 |
|  |  |  | Gender | 0.03 | 0.133 | -0.03, 0.1 | 1.07 | 0.287 | 0.586 |
|  |  |  | Left MT | 0.37 | 0.232 | 0.03, 0.72 | 2.15 | **0.035** | **0.0443** |
|  |  |  | Age (Months) | <.001 | -0.051 | 0.00, 0.00 | -0.46 | 0.649 | 0.771 |
|  |  |  | Scanner Code | 0.096 | 0.313 | 0.03, 0.16 | 2.94 | **0.005** | **0.0221** |
|  |  |  | Worry x Gender | -0.08 | -0.214 | -0.19, 0.03 | -1.52 | 0.135 | 0.328 |
|  |  |  | (Intercept) | 1.84 | NA | 0.77, 2.9 | 3.45 | **0.001** | **0.006** |
| Right Posterior Cingulate Thickness | 75 | 0.3/  0.215 | IUI-B | -0.003 | -0.568 | -0.01, 0 | -3.17 | **0.002** | **0.028** |
|  |  |  | Comm. Int. | 0.11 | 0.392 | 0.02, 0.2 | 2.51 | **0.015** | 0.116 |
|  |  |  | AAp | 0.02 | 0.102 | -0.05, 0.09 | 0.62 | 0.539 | 0.864 |
|  |  |  | Gender | 0.06 | 0.260 | 0.00, 0.12 | 2.10 | **0.040** | 0.240 |
|  |  |  | Right MT | 0.27 | 0.196 | -0.03, 0.57 | 1.80 | 0.077 | 0.084 |
|  |  |  | Age (Months) | <.001 | -0.012 | 0.00, 0.00 | -0.11 | 0.915 | 0.955 |
|  |  |  | Scanner Code | 0.059 | 0.21 | 0.00, 0.12 | 1.89 | 0.063 | 0.189 |
|  |  |  | Worry x Gender | 0.018 | 0.044 | -0.09, 0.13 | 0.32 | 0.750 | 0.900 |
|  |  |  | (Intercept) | 2.05 | NA | -0.3, 2.02 | 4.54 | **<.001** | **<.001** |

Table S7

| IUI - A | | | | | | | | | |
| --- | --- | --- | --- | --- | --- | --- | --- | --- | --- |
| ROI | N | r2/  r2 adj. | IV | Est. | Std. Est. | Conf. Int. | t-value | P | FDR-P |
| Right Caudal Anterior Cingulate Surface Area | 74 | 0.354/  0.307 | IUI-A | 2.63 | 0.22 | 0.19, 5.06 | 2.15 | **0.035** | 0.169 |
|  |  |  | Gender | 8.38 | 0.032 | -59.2, 75.9 | 0.25 | 0.805 | 0.978 |
|  |  |  | Right MSA | 0.01 | 0.622 | 0.01, 0.01 | 4.92 | **<.001** | **<.001** |
|  |  |  | Age (Months) | 0.06 | 0.007 | -1.65, 1.77 | 0.07 | 0.944 | 0.990 |
|  |  |  | Scanner Code | 13.4 | 0.041 | -52.2, 78.9 | 0.41 | 0.685 | 0.783 |
|  |  |  | (Intercept) | -180 | NA | -772, 412 | -0.61 | 0.547 | 0.874 |
| Right Pars Triangularis Surface Area | 78 | 0.244/  0.192 | IUI-A | 3.28 | 0.158 | -1.13, 7.7 | 1.48 | 0.143 | 0.489 |
|  |  |  | Gender | -120 | -0.262 | -243, 2.67 | -1.95 | 0.055 | 0.449 |
|  |  |  | Right MSA | 0.005 | 0.176 | 0.00, 0.01 | 1.32 | 0.192 | 0.192 |
|  |  |  | Age (Months) | -1.46 | -0.114 | -4.25, 1.32 | -1.05 | 0.297 | 0.649 |
|  |  |  | Scanner Code | -113 | -0.20 | -233, 5.9 | -1.89 | 0.062 | 0.248 |
|  |  |  | (Intercept) | 1553 | NA | 520, 2586 | 3.00 | **0.004** | **0.044** |
| Right Lateral Orbitofron-tal Surface Area | 74 | 0.779/  0.763 | IUI-A | -4.88 | -0.20 | -7.77, -1.99 | -3.37 | **0.001** | **0.029** |
|  |  |  | Gender | 0.232 | <.001 | -79.5, 80.0 | 0.005 | 0.995 | 0.995 |
|  |  |  | Right MSA | 0.028 | 0.851 | 0.02, 0.03 | 11.5 | **<.001** | **<.001** |
|  |  |  | Age (Months) | 0.821 | 0.049 | -1.25, 2.89 | 0.79 | 0.431 | 0.807 |
|  |  |  | Scanner Code | 104 | 0.163 | 28.8, 179 | 2.76 | **0.007** | 0.059 |
|  |  |  | (Intercept) | 40.2 | NA | -675, 756 | 0.11 | 0.911 | 0.948 |
| Left Rostral Middle Frontal Surface Area | 74 | 0.822/0.809 | IUI-A | 8.31 | 0.129 | 1.51, 15.11 | 2.44 | **0.017** | 0.139 |
|  |  |  | Gender | -16.4 | -0.012 | -194, 162 | -0.18 | 0.855 | 0.978 |
|  |  |  | Left MSA | 0.08 | 0.924 | 0.07, 0.09 | 14.4 | **<.001** | **<.001** |
|  |  |  | Age (Months) | 1.83 | 0.042 | -2.8, 6.5 | 0.78 | 0.437 | 0.807 |
|  |  |  | Scanner Code | 287 | 0.167 | 106, 468 | 3.17 | **0.002** | **0.028** |
|  |  |  | (Intercept) | -1870 | NA | -3452, -284 | -2.35 | **0.022** | 0.086 |
|  | | | | | | | | | |
| IUI - B | | | | | | | | | |
| ROI | N | r2/  r2 adj. | IV | Est. | Std. Est. | Conf. Int. | t-value | P | FDR-P |
| Left Medial Orbitofrontal Thickness | 74 | 0.224/0.166 | IUI-B | <-.001 | -0.135 | 0.00, 0.00 | -1.20 | 0.234 | 0.561 |
|  |  |  | Gender | 0.003 | 0.013 | -0.05, 0.06 | 0.12 | 0.907 | 0.907 |
|  |  |  | Left MSA | 0.505 | 0.315 | 0.15, 0.86 | 2.84 | **0.006** | **0.008** |
|  |  |  | Age (Months) | -0.001 | -0.118 | 0.00, 0.00 | -1.03 | 0.309 | 0.624 |
|  |  |  | Scanner Code | 0.075 | 0.245 | 0.01, 0.14 | 2.21 | **0.031** | 0.105 |
|  |  |  | (Intercept) | 1.45 | NA | 0.34, 2.55 | 2.61 | **0.011** | **0.040** |
| Right Posterior Cingulate Thickness | 74 | 0.258/0.203 | IUI-B | <-.001 | -0.070 | 0.00, 0.00 | -0.64 | 0.523 | 0.785 |
|  |  |  | Gender | 0.081 | 0.345 | 0.03, 0.13 | 3.17 | **0.002** | **0.028** |
|  |  |  | Right MSA | 0.462 | 0.33 | 0.15, 0.77 | 2.98 | **0.004** | **0.006** |
|  |  |  | Age (Months) | <.001 | 0.071 | 0.00, 0.00 | 0.65 | 0.520 | 0.735 |
|  |  |  | Scanner Code | 0.022 | 0.076 | -0.04, 0.09 | 0.68 | 0.497 | 0.663 |
|  |  |  | (Intercept) | 1.27 | NA | 0.33, 2.21 | 2.69 | **0.009** | **0.040** |

Figure S3

Table S8

| IUI - A | | | | | | | | | |
| --- | --- | --- | --- | --- | --- | --- | --- | --- | --- |
| ROI | N | r2/  r2 adj. | IV | Est. | Std. Est. | Conf. Int. | t-value | P | FDR-P |
| Right Rostral Middle Frontal Surface Area | 36 | 0.855/  0.825 | IUI-A | 16.3 | 0.305 | 5.69, 26.9 | 3.14 | **0.004** | **0.030** |
|  |  |  | Comm. Int. | -33.3 | -0.023 | -310, 244 | -0.25 | 0.808 | 0.885 |
|  |  |  | AAp | 177 | 0.127 | -69.5, 424 | 1.47 | 0.153 | 0.366 |
|  |  |  | Right MSA | 0.09 | 0.830 | 0.07, 0.11 | 10.3 | **<.001** | **<.001** |
|  |  |  | Age (Months) | -1.79 | -0.043 | -8.35, 4.77 | -0.56 | 0.581 | 0.730 |
|  |  |  | Scanner Code | 152 | 0.080 | -140, 444 | 1.06 | 0.296 | 0.789 |
|  |  |  | (Intercept) | -2093 | NA | -4562, 376 | -1.73 | 0.094 | 0.423 |
| Right Medial Orbitofrontal Surface Area | 36 | 0.768/  0.72 | IUI-A | 6.52 | 0.421 | 2.69, 10.34 | 3.49 | **0.002** | **0.023** |
|  |  |  | Comm. Int. | 18.1 | 0.049 | -68, 105 | 0.43 | 0.672 | 0.8851 |
|  |  |  | AAp | -40.7 | -0.108 | -119, 38 | -1.05 | 0.301 | 0.4621 |
|  |  |  | Right MSA | 0.022 | 0.716 | 0.02, 0.03 | 7.32 | **<.001** | **<.001** |
|  |  |  | Age (Months) | 0.648 | 0.076 | -1.03, 2.33 | 0.788 | 0.437 | 0.7303 |
|  |  |  | Scanner Code | -246 | -0.346 | -380, -111 | -3.741 | **<.001** | **0.010** |
|  |  |  | (Intercept) | -200 | NA | -919, 520 | -0.567 | 0.5748 | 0.8456 |

Table S9

| IUI - A | | | | | | | | | |
| --- | --- | --- | --- | --- | --- | --- | --- | --- | --- |
| ROI | N | r2/  r2 adj. | IV | Est. | Std. Est. | Conf. Int. | t-value | P | FDR-P |
| Right Rostral Middle Frontal Surface Area | 38 | 0.81/  0.787 | IUI-A | 16.7 | 0.305 | 8.03, 25.4 | 3.92 | **<.001** | **0.005** |
|  |  |  | Right MSA | 0.103 | 0.881 | 0.08, 0.12 | 11.3 | 0 | 0 |
|  |  |  | Age (Months) | 0.84 | 0.020 | -5.81, 7.48 | 0.26 | 0.800 | 0.903 |
|  |  |  | Scanner Code | 235 | 0.116 | -87.48, 556.74 | 1.48 | 0.148 | 0.394 |
|  |  |  | (Intercept) | -3475 | NA | -5767, -1182 | -3.08 | **0.004** | **0.049** |
| Right Medial Orbitofrontal Surface Area | 35 | 0.74/  0.706 | IUI-A | 6.78 | 0.459 | 3.95, 9.61 | 4.89 | **<.001** | **<.001** |
|  |  |  | Right MSA | 0.021 | 0.708 | 0.02, 0.03 | 7.53 | **<.001** | **<.001** |
|  |  |  | Age (Months) | 0.53 | 0.065 | -1.05, 2.11 | 0.68 | 0.501 | 0.8587 |
|  |  |  | Scanner Code | -150 | -0.163 | -328, 27.3 | -1.73 | .0900 | 0.3941 |
|  |  |  | (Intercept) | -178 | NA | -803, 448 | -0.58 | 0.566 | 0.7874 |

Table S10

| IUI - B | | | | | | | | | |
| --- | --- | --- | --- | --- | --- | --- | --- | --- | --- |
| ROI | N | r2/  r2 adj. | IV | Est. | Std. Est. | Conf. Int. | t-value | P | FDR-P |
| Left Rostral Anterior Cingulate Surface Area | 36 | 0.745/0.693 | IUI-B | -4.67 | -0.691 | -7.06, -2.28 | -4.00 | **<.001** | **0.010** |
|  |  |  | Comm. Int. | 79.54 | 0.249 | -3.86, 163 | 1.95 | 0.061 | 0.441 |
|  |  |  | AAp | 106.5 | 0.495 | 44.2, 169 | 3.50 | **0.002** | **0.036** |
|  |  |  | Left MSA | 0.013 | 0.838 | 0.01, 0.02 | 8.53 | **<.001** | **<.001** |
|  |  |  | Age (Months) | 1.70 | 0.225 | 0.07, 3.32 | 2.14 | **0.041** | 0.260 |
|  |  |  | Scanner Code | 39.4 | 0.155 | -10.8, 89.6 | 1.61 | 0.119 | 0.568 |
|  |  |  | (Intercept) | -400 | NA | -912, 112 | -1.60 | 0.121 | 0.500 |

Table S11

| IUI - B | | | | | | | | | |
| --- | --- | --- | --- | --- | --- | --- | --- | --- | --- |
| ROI | N | r2/  r2 adj. | IV | Est. | Std. Est. | Conf. Int. | t-value | P | FDR-P |
| Left Rostral Anterior Cingulate Surface Area | 37 | 0.559/  0.503 | IUI-B | -0.703 | -0.108 | -2.29, 0.89 | -0.901 | 0.375 | 0.776 |
|  |  |  | Left MSA | 0.012 | 0.758 | 0.01 - 0.02 | 6.29 | **<.001** | **<.001** |
|  |  |  | Age (Months) | 1.58 | 0.192 | -0.47 - 3.63 | 1.57 | 0.126 | 0.336 |
|  |  |  | Scanner Code | 23.11 | 0.088 | -40.0, 86.3 | 0.745 | 0.462 | 0.865 |
|  |  |  | (Intercept) | -524 | NA | -1160, 111 | -1.68 | 0.103 | 0.493 |

Table S12

| IUI - A | | | | | | | | | |
| --- | --- | --- | --- | --- | --- | --- | --- | --- | --- |
| ROI | N | r2/  r2 adj. | IV | Est. | Std. Est. | Conf. Int. | t-value | P | FDR-P |
| Left Rostral Middle Frontal  Surface Area | 39 | 0.867/  0.842 | IUI-A | -11.6 | -0.200 | -22.66, -0.47 | -2.12 | **0.041** | 0.263 |
|  |  |  | Comm. Int. | -70.1 | -0.033 | -359, 219 | -0.49 | 0.625 | 0.715 |
|  |  |  | AAp | 115.6 | 0.120 | -57.3, 288 | 1.36 | 0.183 | 0.637 |
|  |  |  | Left MSA | 0.091 | 0.911 | 0.08, 0.1 | 13.54 | **<.001** | **<.001** |
|  |  |  | Age (Months) | 0.780 | 0.119 | -0.1, 1.66 | 1.81 | 0.080 | 0.424 |
|  |  |  | Scanner Code | 208 | 0.157 | 28.41, 388 | 2.36 | **0.025** | 0.197 |
|  |  |  | (Intercept) | -2260 | NA | -3650, -871 | -3.31 | **0.002** | **0.018** |

Table/Figure Captions for Supplementary

Figure S1. Cortical Regions of Interest For the Current Study

Figure S2. Non-cortical Regions of Interest For the Current Study

Table S1. Correlations of Predictors Across Time Points in the Adult Sample (top) and in the Adolescent Sample (bottom)

Note: p< .0001 ****, p<.001 ***, p<.01 **, p< .05 *, p< .1 •; Intolerance of Uncertainty Index subscale A and B (IUI-A/B), Anxious Apprehension-specific factor score (AAp), Common Internalizing factor score (Comm. Int.), Time point 1 (TP1), Time point 2 (TP2)

Table S2. Correlations of Brain Morphometric Measures Across Time Points in the Adult Sample (top) and the Adolescent Sample (bottom)

Note: p< .0001 ****, p<.001 ***, p<.01 **, p< .05 *, p< .1 •; Mean Surface Area (MSA), Mean Thickness (MT), Estimated Intracranial Volume (EIV), Timepoint 1 (TP1), Timepoint 2 (TP2)

Table S3. Correlations of Predictor Variables of Interest and Whole-Brain Measures in the Adult Sample (Top) and the Adolescent Sample (Bottom)

Note: p< .0001 ****, p<.001 ***, p<.01 **, p< .05 *, p< .1 • ; Averaged (Avg.), Intolerance of Uncertainty Index subscale A and B (IUI-A/B), Anxious Apprehension-specific factor score (AAp), Common Internalizing factor score (Comm. Int.), Estimated Intracranial Volume (EIV), Mean Surface Area (MSA), Mean Thickness (MT)

Table S4. Outputs for Models Including IUI-A or IUI-B and Internalizing Factor Scores in the Adult Sample.

Bolded values are significant at p < .05 or FDR-p<.05.; Region of Interest (ROI), r2 adjusted (r2 adj.), Independent Variable (IV), Estimate (Est.) Standardized Estimate (Std. Est.), Confidence Interval (Conf. Int.), Intolerance of Uncertainty Index subscale A and B (IUI-A/B), Common Internalizing factor score (Comm. Int.), Anxious Apprehension-specific factor score (AAp), Mean Surface Area (MSA), Estimated Intracranial Volume (EIV)

Table S5. Outputs for Models Including Only IUI-A or IUI-B in the Adult Sample.

Bolded values are significant at p < .05 or FDR-p<.05.; Region of Interest (ROI), r2 adjusted (r2 adj.), Independent Variable (IV), Estimate (Est.) Standardized Estimate (Std. Est.), Confidence Interval (Conf. Int.), Intolerance of Uncertainty Index subscale A and B (IUI-A/B), Mean Surface Area (MSA), Estimated Intracranial Volume (EIV)

Table S6. Outputs for Models Including IUI-A or IUI-B and Internalizing Factor Scores in the Adolescent Sample.

Bolded values are significant at p < .05 or FDR-p<.05.; Region of Interest (ROI), r2 adjusted (r2 adj.), Independent Variable (IV), Estimate (Est.) Standardized Estimate (Std. Est.), Confidence Interval (Conf. Int.), Intolerance of Uncertainty Index subscale A and B (IUI-A/B), Common Internalizing factor score (Comm. Int.), Anxious Apprehension-specific factor score (AAp), Mean Surface Area (MSA), Mean Thickness (MT)

Table S7. Outputs for Models Including Only IUI-A or IUI-B in the Adolescent Sample.

Bolded values are significant at p < .05 or FDR-p<.05.; Region of Interest (ROI), r2 adjusted (r2 adj.), Independent Variable (IV), Estimate (Est.) Standardized Estimate (Std. Est.), Confidence Interval (Conf. Int.), Intolerance of Uncertainty Index subscale A and B (IUI-A/B), Mean Surface Area (MSA), Mean Thickness (MT)

Figure S3. Residual Associations of IUI-A and IUI-B with Characteristics of Brain Morphology in Gender Specific Samples.

Shown at the top are the associations between the residuals of IUI-A and the residuals of the a) right rostral middle frontal surface area and b) right medial orbitofrontal surface area in the female adolescents. Shown at the bottom are the associations of IUI-B residuals and the residuals of the surface area of the c) left rostral anterior cingulate. The grey shaded regions on the graphs are the 95% confidence intervals. Residuals were calculated controlling for Common Internalizing, Anxious Apprehension, Age, Scanner Code, and Overall Brain Characteristic (e.g., Mean Hemispheric Surface Area).

Table S8. Outputs for Models Including IUI-A and Internalizing Factor Scores in the Female-Only Adolescent Sample.

Bolded values are significant at p < .05 or FDR-p<.05.; Region of Interest (ROI), r2 adjusted (r2 adj.), Independent Variable (IV), Estimate (Est.) Standardized Estimate (Std. Est.), Confidence Interval (Conf. Int.), Intolerance of Uncertainty Index subscale A and B (IUI-A/B), Common Internalizing factor score (Comm. Int.), Anxious Apprehension-specific factor score (AAp), Mean Surface Area (MSA)

Table S9. Outputs for Models Including Only IUI-A in the Female-Only Adolescent Sample.

Bolded values are significant at p < .05 or FDR-p<.05.; Region of Interest (ROI), r2 adjusted (r2 adj.), Independent Variable (IV), Estimate (Est.) Standardized Estimate (Std. Est.), Confidence Interval (Conf. Int.), Intolerance of Uncertainty Index subscale A and B (IUI-A/B), Mean Surface Area (MSA)

Table S10. Outputs for Models Including IUI-A or IUI-B and Internalizing Factor Scores in the Male-Only Adolescent Sample.

Bolded values are significant at p < .05 or FDR-p<.05.; Region of Interest (ROI), r2 adjusted (r2 adj.), Independent Variable (IV), Estimate (Est.) Standardized Estimate (Std. Est.), Confidence Interval (Conf. Int.), Intolerance of Uncertainty Index subscale A and B (IUI-A/B), Common Internalizing factor score (Comm. Int.), Anxious Apprehension-specific factor score (AAp), Mean Surface Area (MSA)

Table S11. Outputs for Models Including Only IUI-A or IUI-B in the Male-Only Adolescent Sample.

Bolded values are significant at p < .05 or FDR-p<.05.; Region of Interest (ROI), r2 adjusted (r2 adj.), Independent Variable (IV), Estimate (Est.) Standardized Estimate (Std. Est.), Confidence Interval (Conf. Int.), Intolerance of Uncertainty Index subscale A and B (IUI-A/B), Mean Surface Area (MSA)

Table S12. Model Outputs for the Left Rostral Middle Frontal Surface Area with IUI-A and Internalizing Covariates in the Adult Sample.

Bolded values are significant at p < .05 or FDR-p<.05.; Region of Interest (ROI), r2 adjusted (r2 adj.), Independent Variable (IV), Estimate (Est.) Standardized Estimate (Std. Est.), Confidence Interval (Conf. Int.), Intolerance of Uncertainty Index subscale A and B (IUI-A/B), Common Internalizing factor score (Comm. Int.), Anxious Apprehension-specific factor score (AAp), Mean Surface Area (MSA)
